# Semisynthetic prion protein (PrP) variants carrying glycan mimics at position 181 and 197 do not form fibrils†
†Electronic supplementary information (ESI) available. See DOI: 10.1039/c7sc02719b



**DOI:** 10.1039/c7sc02719b

**Published:** 2017-07-24

**Authors:** Can Araman, Robert E. Thompson, Siyao Wang, Stefanie Hackl, Richard J. Payne, Christian F. W. Becker

**Affiliations:** a Institute of Biological Chemistry , Department of Chemistry , University of Vienna , Waehringer Strasse 38 , 1090 , Vienna-AT , Austria . Email: christian.becker@univie.ac.at; b School of Chemistry , The University of Sydney , Sydney , NSW 2006 , Australia

## Abstract

The prion protein (PrP) is an *N*-glycosylated protein attached to the outer leaflet of eukaryotic cell membranes *via* a glycosylphosphatidylinositol (GPI) anchor. Different prion strains have distinct glycosylation patterns and the extent of glycosylation of potentially pathogenic misfolded prion protein (PrP^Sc^) has a major impact on several prion-related diseases (transmissible spongiform encephalopathies, TSEs). Based on these findings it is hypothesized that posttranslational modifications (PTMs) of PrP influence conversion of cellular prion protein (PrP^C^) into PrP^Sc^ and, as such, modified PrP variants are critical tools needed to investigate the impact of PTMs on the pathogenesis of TSEs. Here we report a semisynthetic approach to generate PrP variants modified with monodisperse polyethyleneglycol (PEG) units as mimics of N-glycans. Incorporating PEG at glycosylation sites 181 and 197 in PrP induced only small changes to the secondary structure when compared to unmodified, wildtype PrP. More importantly, *in vitro* aggregation was abrogated for all PEGylated PrP variants under conditions at which wildtype PrP aggregated. Furthermore, the addition of PEGylated PrP as low as 10 mol% to wildtype PrP completely blocked aggregation. A similar effect was observed for synthetic PEGylated PrP segments comprising amino acids 179–231 alone if these were added to wildtype PrP in aggregation assays. This behavior raises the question if large N-glycans interfere with aggregation *in vivo* and if PEGylated PrP peptides could serve as potential therapeutics.

## Abstract 2

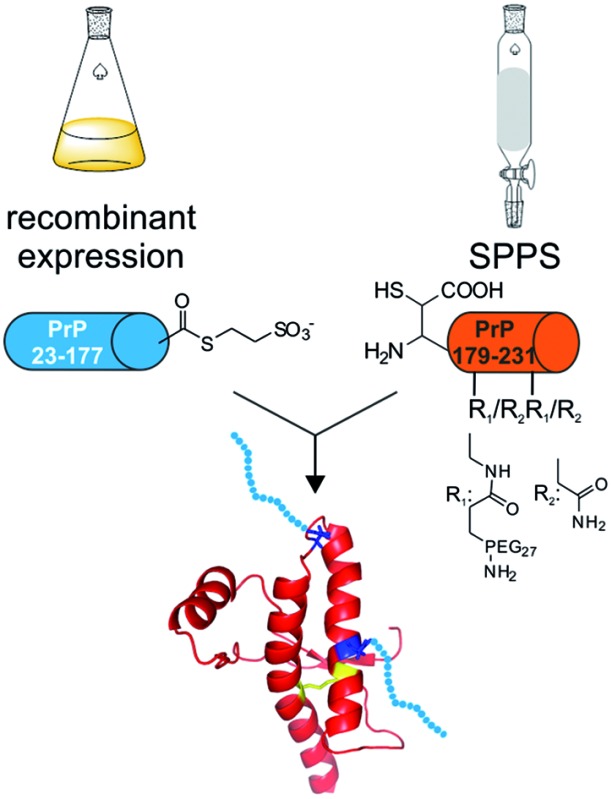
Semisynthesis and characterization of homogeneously mono- and di-PEGylated full length PrP variants to study the impact of PEGylation (as *N*-glycan mimics) on protein folding and aggregation.

## Introduction

Prion diseases, also known as transmissible spongiform encephalopathies (TSE), are a class of infectious, progressive and fatal neurodegenerative disorders associated with the loss of cognitive skills and neuronal dysfunction in animals and humans.^1,2^ Accumulation of misfolded proteinaceous particles (prions) is regarded a hallmark feature that is necessary for progression to TSEs.^3^ However, it is still not entirely understood how these aggregates are formed and when or why the conversion of cellular, non-pathogenic prion protein (PrP^C^) into pathogenic scrapie PrP (PrP^Sc^) occurs. PrP^C^ is bound to the outside of the plasma membrane *via* a glycosylphosphatidyl-inositol (GPI) anchor and is *N*-glycosylated either on one or two asparagine residue (Asn 181 or 197).^4^ The impact of the GPI anchor on the conversion of PrP^C^ into PrP^Sc^ has been widely investigated and there is strong evidence that anchoring to the plasma membrane is crucial for this process.^5–7^ *N*-glycosylation of PrP has been reported by Collinge and coworkers as a marker for distinguishing TSEs by comparing the glycosylation patterns of PrP in variant Creutzfeld–Jakob Disease (vCJD) and sporadic CJD patients.^8^ In 2008, Gambetti *et al.* described the discovery of a new sporadic TSE, protease-sensitive prionopathy (PSPr). In PSPr, similar to familial CJDV180I, the absence of di-glycosylated PrP^Sc^ was reported. This finding suggests some selectivity in the process of conversion of glycosylated and non-glycosylated PrP^C^ into PrP^Sc^.^9^ Additional studies from the same group showed that not only di-glycosylated PrP^Sc^, but also mono-glycosylated PrP^C^ (at Asn 181), was unable to be converted into PrP^Sc^.^10^

These results provide strong evidence for the impact of glycosylation on prion formation and transmissibility. However, until now unequivocal proof for the influence of *N*-glycosylation on prion pathogenesis has not been forthcoming, due in major part to the heterogeneity of glycosylation patterns found in PrP from various sources.^11,12^ Thus, strategies to generate homogeneous PrP preparations with defined glycosylation patterns are urgently needed.

**Scheme 1:**
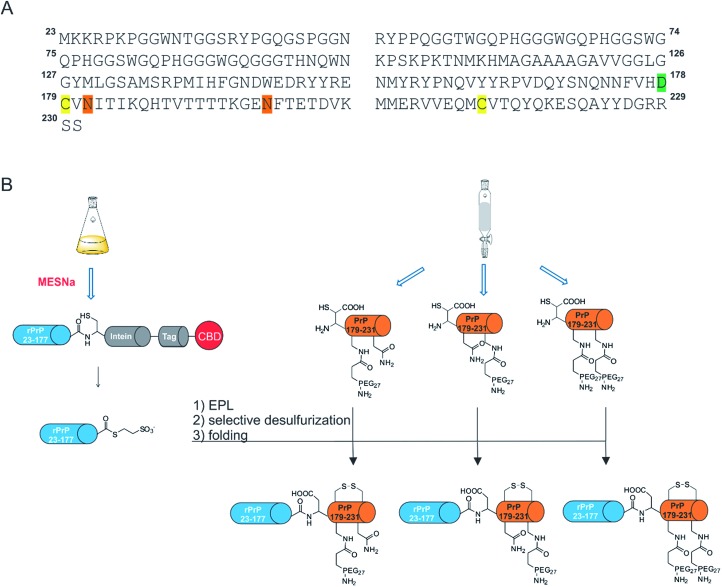
Semisynthesis of homogeneously mono- and di-PEGylated full length PrP variants. MESNa: 2-mercaptoethanesulfonate; EPL: Expressed Protein Ligation, rPrP: recombinant prion protein. (A) Primary sequence of full length PrP. Modifications are highlighted in different colors; green: β-mercapto-aspartate, orange: N→Dpr (l-diaminopropionic acid) mutations, yellow: C179 and C214. (B) Schematic representation of the EPL-desulfurization approach. Blue cylinders: recombinant C-terminally truncated PrP either fused to MxeGyr-Intein (gray) or as a protein α-thioester. Orange cylinders: synthetic C-terminal PrP comprising amino acids 178–231 with PEG modifications.

In an attempt to address this question, we expanded on our previously developed semisynthesis strategy to access membrane-anchored, full length (FL) PrP variants^13–15^ to include modifications at positions 181 and 197 (Scheme 1). Our strategy centered on the use of native chemical ligation (NCL) and expressed protein ligation (EPL)^16,17^ methods that enable the synthesis of a variety of different proteins^18,19^ carrying site-specific modifications.^20,21^ To test the impact of large N-glycan structures at positions 181 and 197 in PrP, we prepared semisynthetic PrP variants with monodisperse polyethylene glycol (PEG) units as glycan surrogates. Homogeneous PEG has been previously shown to serve as an effective glycan mimic and we rationalized that the incorporation of PEG would facilitate rapid access to the desired protein variants in this work.^22–24^ Selection of the PEG moiety was based on the availability of appropriately functionalized molecules and, more importantly, on the previously reported glycan structures found on PrP with molecular weights greater than 1500 Da.^11,12^ The carboxyl-functionalized PEG_27_ fits these criteria well and therefore we hypothesized that it would be a good mimic of the native glycan.

A major obstacle for our new semisynthetic strategy was the lack of suitably placed cysteine residues to efficiently link synthetic and recombinant PrP segments (Scheme 1A). Several methods have been described to overcome this challenge, *e.g.* replacing a native alanine residue with a cysteine for use in native chemical ligation, followed by subsequent radical desulfurization back to alanine, as well as using unnatural amino acids bearing β-, γ- or δ-sulfhydryl moieties that can be employed as cysteine surrogates in native chemical ligation.^25–29^ Due to the presence of two crucial cysteine residues in PrP (to form a stabilizing disulfide bridge) we chose β-mercapto-aspartate at position 178 as the ligation site, as it has been shown that this β-thiol amino acid can be selectively desulfurized in the presence of unprotected cysteine residues (Scheme 1B).^30^ Based on this strategy, we report the successful synthesis of mono- and di-PEGylated full length PrP variants to study the impact of PEGylation (as a N-glycan mimic) on protein folding and aggregation. Specifically, we show that the use of EPL in combination with selective desulfurization reactions gave rise to the first homogeneously mono- and di-PEGylated full length PrP variants.

## Results and discussion

### Generation of recombinant PrP 23-177 MESNa-thioester

**Fig. 1:**
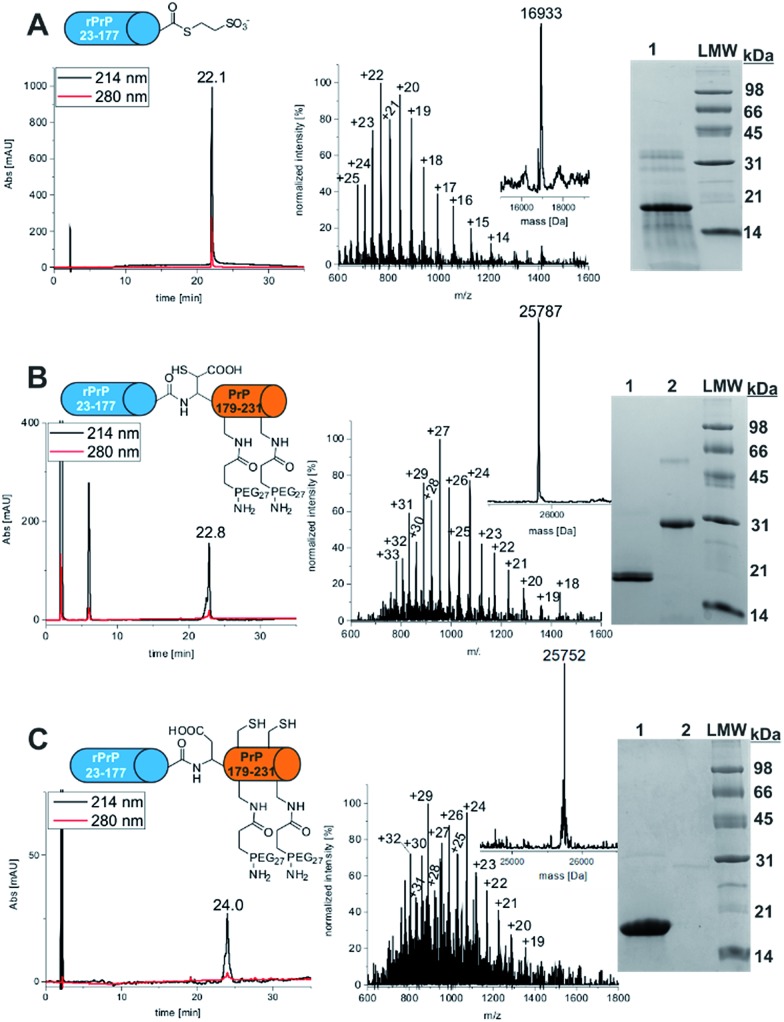
Synthesis of FL PrP-181 & 197PEG_27_. (A) Final analysis of PrP 23-177-MESNA thioester *via* analytical RP-HPLC (linear gradient 5–65% ACN in 30 min), ESI-MS (expected mass: 16 931.6 Da, observed mass: 16 933 Da) and SDS-PAGE (lane 1: PrP 23-177-MESNA thioester, lane 2: LMW); (B) characterization of FL-PrP-178 β-mercapto-Asp-181 & 197PEG_27_ *via* RP-HPLC, ESI-MS (expected mass: 25 786.6 Da, observed mass: 25 787 Da) and SDS-PAGE (lane 1: PrP 23-177-MESNA thioester, lane 2: FL-PrP-178 β-mercapto-Asp-181 & 197PEG_27_); (C) characterization of FL-PrP-181 & 197PEG_27_ *via* RP-HPLC, ESI-MS (expected mass: 25 754.6 Da, observed mass: 25 752 Da) and SDS-PAGE (lane 1: PrP 23-177-MESNA thioester, lane 2: FL-PrP-181 & 197PEG_27_).

Recombinant PrP α-thioester comprising amino acids 23-177 was generated from a PrP 23-177-MxeIntein-His_6_-CBD fusion construct cloned into a previously described pTXB3 plasmid (Fig. 1).^15^ The fusion protein was isolated from *E. coli* using Ni-affinity chromatography and subsequently cleaved in the presence of MESNa within 24 h. PrP 23-177 α-thioester was purified by preparative RP-HPLC and characterized *via* analytical RP-HPLC, ESI-MS and SDS-PAGE (Fig. 1A). The purified product was obtained with a yield of 11 mg protein thioester per L culture and in high purity (>95%).

### SPPS of mono-, di-PEGylated and acetylated PrP peptides comprising amino acids 178–231

To enable traceless NCL reactions, we introduced the unnatural amino acid β-mercapto aspartate (β-mercapto-Asp) at the N-terminus of all six PrP peptide variants used here.^30^ Three of these peptides were PEGylated by introducing l-diaminopropionic acid (Dpr) carrying an orthogonal Mtt side chain protecting group at either one or both Asn sites (181 & 197). These residues were selectively deprotected and reacted with Fmoc-PEG_27_-COOH (Fig. S1–S3†). Another set of three peptides containing l-Dpr(Mtt)-OH were *N*-acetylated to provide controls for the impact of Asn to Dpr mutations and for PEG attachment on folding and aggregation (Fig. S4–S6†). All synthetic peptides showed a shoulder or peak broadening in their HPLC traces (Fig. S1–S3,† *t* = 24 min). This was owing to the use of β-mercapto-Asp that was diastereomeric at the β-position leading to peptide epimers. As the chiral center at the β-carbon is removed upon desulfurization, epimer formation is of no consequence for the final products. All peptides were obtained in good yields and purity (Tables S1 and S2†).

### Expressed protein ligation of recombinant PrP 23-177 MESNa-thioester with mono- & di-PEGylated or acetylated peptides

Ligation of PrP α-thioesters with either PEGylated or acetylated PrP peptides was achieved at concentrations of 1.2 mM of protein thioester and 1 mM of PEGylated peptide in the presence of a thiol additive (MPAA) for two hours in a reaction volume of 200–1000 μL. Reaction progress was monitored *via* analytical RP-HPLC (Fig. S7–S11†). Reactant conversions varied between 90–95% within 2 h, after which all ligation products were purified, lyophilized and analyzed *via* analytical RP-HPLC, ESI-MS and SDS-PAGE (see Fig. 1B for di-PEGylated product, Fig. S8 and S10† for mono-PEGylated products and Fig. S22† for mono-acetylated PrP variant). Mono- and di-PEGylated, full length PrP variants were obtained in good purity (>90–95%) in moderate to high yields (11–78%).

### Selective desulfurization

To selectively desulfurize β-mercapto-Asp in mono- and di-PEGylated full length PrP without concomitant desulfurization of the native cysteine residues in position 179 and 214, a protocol described by Thompson *et al.* was followed.^30^ Full length PrP variants were dissolved in desulfurization buffer (6 M Gdn-HCl, 200 mM NaPi, 250 mM TCEP, 50 mM DTT, pH 2.8) with excess DTT under rigorous shaking at 66 °C. The reaction progress was monitored by LC-MS and crude reaction mixtures were purified by RP-HPLC. Selectively desulfurized FL-PrP-181PEG_27_ (Fig. S12†), FL-PrP197PEG_27_ (Fig. S13†) and FL-PrP-181 & 197PEG_27_ (Fig. 1C) were analyzed by analytical RP-HPLC, ESI-MS, SDS-PAGE and obtained in yields of 57–75% as single peaks without shoulders (Table 1). In the HPLC traces of all products, only a single peak was detected.

**Table 1:** Synthesis and folding yields of PEGylated PrP variants

Variant	Amount	Synthesis yield	Folding yield
FL-PrP-181PEG_27_	1.6 mg	60%	85%
FL-PrP-197PEG_27_	1.8 mg	75%	63%
FL-PrP-181 & 197PEG_27_	1.5 mg	57%	74%

### Folding of PEGylated and acetylated PrP variants

Recombinantly produced PrP as well as semisynthetic variants are often (partly) denatured and require (re-)folding steps,^13,15,31^ which also holds true for all PrP variants generated in this work. We chose a stepwise dilution strategy for folding of wt, PEGylated and acetylated PrP variants, in which we reduced the concentration of Gdn-HCl from 6 to 2 M in 50 mM Tris–HCl buffer (pH 8.0). Dilution was achieved with refolding buffer and a subsequent dialysis step against the refolding buffer (20 mM NaOAc, pH 5.0) was included to reduce Gdn-HCl below 2 mM. Reduced and oxidized glutathione (3 mM : 0.3 mM) was added to the refolding buffer to facilitate disulfide formation between C179 and C214 as previously described by us and others.^15,32,33^ Typical yields for folding of PEGylated PrP variants are summarized in Table 1. To exclude the possibility that mixed disulfides with reduced glutathione (GSH) are formed during the folding process, folded samples were analyzed *via* LC-MS.

### Circular dichroism (CD) spectroscopy of PrP variants

Previous work from our group indicated that semisynthetic lipidated and non-lipidated PrP variants adopt a predominantly α-helical structure.^13–15^ This finding is in good agreement with the fact that PrP^C^ and recombinant PrP share a common α-helix-rich secondary structure.^34^ It has been shown that PEG units can increase the solubility and conformational stability of proteins such as EPO and G-CSF^22,35^ but do not induce significant changes in secondary structure.^36^ CD measurements of our newly available PEGylated PrP variants revealed that the α-helical content was decreased in comparison to wt FL-PrP and unmodified, semisynthetic FL-PrP (Table 2, Fig. S14†).^37^ Mono-PEGylated PrP variants FL-PrP-181PEG_27_ (Fig. S14,† blue diamonds) and FL-PrP-197PEG_27_ (Fig. S14,† black squares) exhibit 11.3 and 9.4% less α-helicity, respectively, whereas di-PEGylated PrP (Fig. S14,† magenta dots) showed a dramatic decrease of 25.7% in helicity. Mono-PEGylated PrP variants showed only a slight increase in β-sheet and β-turn content compared to wt FL-PrP. In contrast, a very high β-sheet content (31.3%) was measured for di-PEGylated PrP. This observation can be of interest because PEGylation (as a mimic of large glycans) could induce different folding states that are either on the pathway towards conversion into β-sheet rich aggregated PrP forms (*e.g.* PrP^Sc^) or resemble off-pathway states that protect from aggregation. Due to the lack of homogeneous glycosylated PrP preparations to date, only mixtures of different mono- and di-glycosylated PrP variants isolated from eukaryotic sources have been analyzed. In 2008 Gerwert and coworkers reported structural changes of native PrP^C^ isolated from Syrian hamster brains (ShaPrP^C^).^34^ In this study, a mixture of different posttranslationally modified PrP^C^ isoforms exhibited a similar secondary structure as recombinantly produced, fully unmodified PrP. The α-helical content was 28%, β-sheet 9%, β-turn 7% and random coil 57%. Our results for homogeneously mono-PEGylated PrP variants showed a similar α-helicity when compared to ShaPrP^C^ (Table 2), but the content of β-sheet was slightly higher for FL-PrP-181PEG_27_ (8.2%) and FL-PrP-197PEG_27_ (2.5%). Di-PEGylated PrP exhibits a much higher degree of β-sheet than SHaPrP^C^. However, comparing a complex mixture of differently modified native PrP variants analyzed by IR with homogeneous, mono- and di-PEGylated PrP analyzed by CD is difficult, as the observed differences could be attributed to deviations in secondary structure of individual species, the analysis technique, PEGylated Dpr as an N-glycan mimic or combinations thereof. In order to assess if changing Asn to Dpr for PEG attachment has an effect on the secondary structure of FL-PrP, we utilized CD spectroscopy with folded FL-PrP-178β-mercapto-Asp-197Dpr(NHAc). This variant adopted a predominantly α-helical structure (Fig. S20†) similar to that of wt FL-PrP. These results suggest that PEGylation and not the change of Asn to Dpr is the driving force behind the conformational change of PEGylated PrP variants.

**Table 2:** Distribution of secondary structure elements among different PrP variants [%]^*a*^

PrP variant	α-Helical	Antiparallel^*b*^	Parallel^*c*^	β-Turn	r.c.
FL-PrP-181PEG_27_	27.3	9.9	7.3	15.3	40.3
FL-PrP-197PEG_27_	28.6	6.8	4.7	21.6	38.6
FL-PrP-181 & 197PEG_27_	12.3	26.1	5.2	20.8	37.6
wt FL-PrP	38.0	8.0	5.9	16.3	31.8

^*a*^All values are presented in %.

^*b*^Antiparallel β-sheet content.

^*c*^Parallel β-sheet content. r.c. = random coil, wt = wild type.

Taken together, the α-helicity of PEGylated PrP variants was comparable with that of fully processed PrP^C^ from hamster cells, whereas the β-turn content was increased in PEGylated PrP variants. The latter could hint towards an increased tendency to form PrP aggregates and even fibrils as these consist of β-sheet rich PrP.

To test if our synthetic PrP peptides comprising amino acids 178–231 have a preferred secondary structure, we performed CD measurements using mono- and di-PEGylated PrP peptides as well as peptides with acetylated Dpr side chains. To the best of our knowledge, shorter PrP peptides within this domain (aa 173–195)^38^ and other neighboring domains (aa 125–170, 142–170 and 156–170) possess random coiled structures under physiological conditions (in water or biological buffers at pH 3–6).^39^ As described by Ronga *et al.*, addition of trifluoroethanol induces the adoption of mainly α-helical secondary structures for short PrP peptides (aa 173–195).^38^ Interestingly, PEGylated PrP peptides (aa 178–231) possess more α-helical elements than their full length counterparts (Fig. S17–S19†). PrP peptides (aa 178–231) carrying acetyl groups on Dpr residues in positions 181 or 197, respectively, showed a predominantly random coil secondary structure (Fig. S15 and S16†), indicating that PEGylation impacts folding of these peptides by stabilizing secondary structure elements. Recent studies with PEGylated peptides and proteins showed that the size and site of PEG attachment can play an important role in stabilization of native structures.^23,24,40,41^ Even though there is literature evidence that PEGylated proteins retain their native-like folding and PEGylation provides an increase in conformational stability,^40,42,43^ Natalello *et al.* reported a decrease in conformational stability and slight increase in aggregation sensitivity of PEGylated G-CSF.^44^ Furthermore, PEGylated hen egg white lysozyme, featured a minor loss of α-helical content compared to wt lysozyme,^45^ making any rational generalized conclusions about the effect of PEGylation on peptide and protein secondary structure very challenging. Therefore, a case by case analysis is required for each PEGylated species as different effects of PEGylation on structure are observed in full length PrP (less helicity and more β-sheet) when compared to the C-terminal peptides (increased helicity) and non-PEGylated PrP.

### *In vitro* aggregation assays with modified PrP variants

A hallmark of prion disease is the formation of amyloid fibrils. ThT aggregation assays are a well-established method to measure the degree of protein aggregation *in vitro*.^46^

**Fig. 2:**
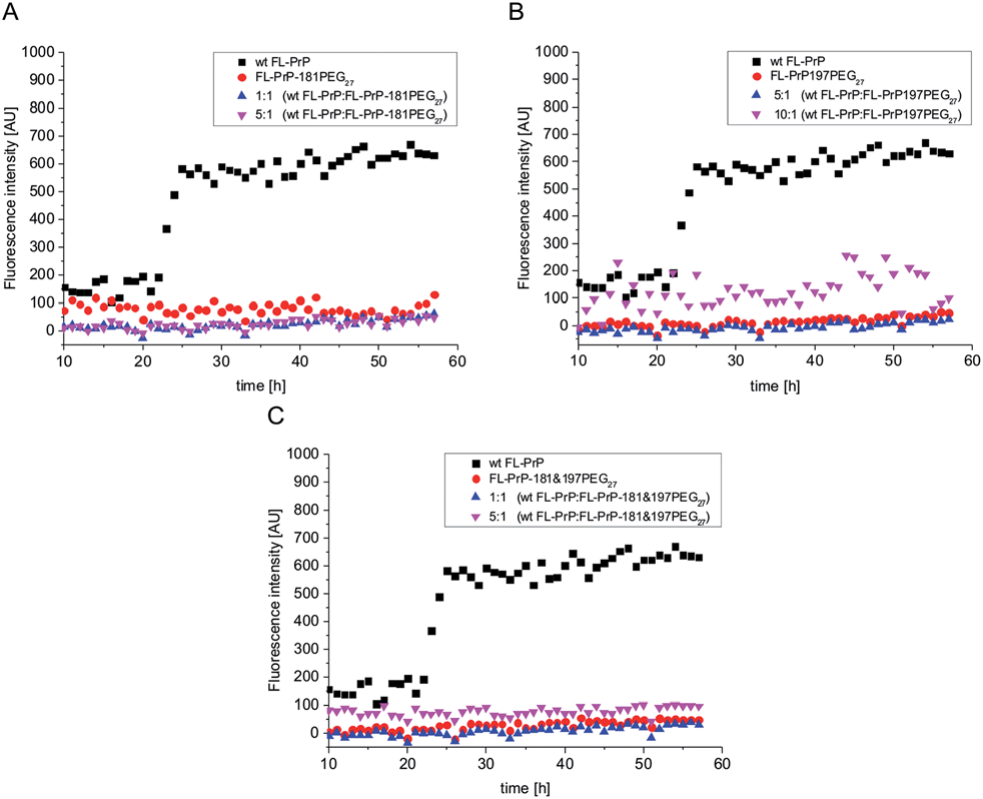
*In vitro* aggregation assays of non-, mono- and di-PEGylated PrP variants. Black squares: wild type PrP; red circles: PEGylated, semisynthetic PrP variants; magenta and blue triangles: mixtures of PEGylated PrP with wild type PrP.

**Fig. 3:**
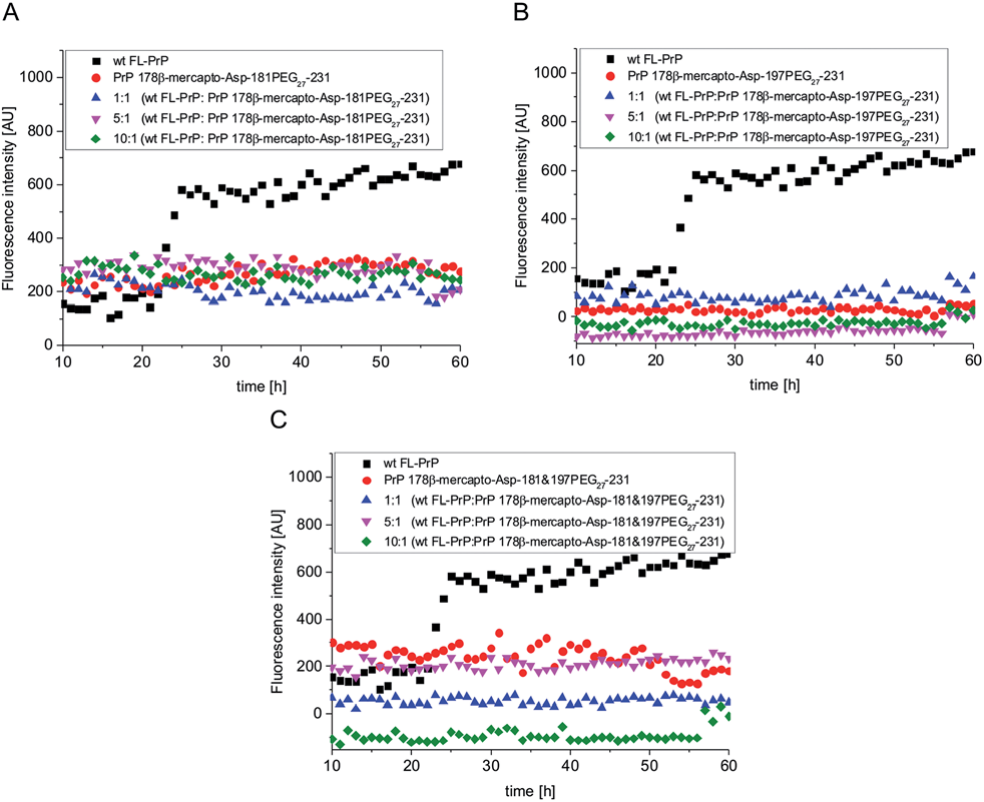
*In vitro* aggregation assays of mono- and di-PEGylated PrP peptides. Black squares: wild type PrP (positive control); red circles: PEGylated, semisynthetic PrP variants; blue triangles: mixture of PEGylated PrP with wild type PrP; magenta triangles: mixture of PEGylated PrP with a 5-fold excess of wild type PrP; green diamonds: mixture of PEGylated PrP with a 10-fold excess of wild type PrP.

For aggregation assays with modified PrP variants, we followed a protocol described by Breydo *et al.*.^47^ Briefly, folded, PEGylated PrP variants as well as one *N*-acetylated variant were dissolved at a concentration of 4 μM (0.1 mg ml^–1^) in 2 M Gdn-HCl buffer with 50 mM Tris–HCl at pH 6. Bovine Serum Albumin (BSA) and wt FL-PrP were used as negative and positive controls, respectively. Remarkably, we observed that wt FL-PrP (Fig. 2, black squares) started to aggregate after ∼20 h but PEGylated PrP variants did not show any fibril formation (traceable by no increase of ThT fluorescence in Fig. 2A–C, red circles). We hypothesize that this effect is caused by the large PEG_27_ modification preventing fibril formation by steric hindrance or *via* its impact on secondary structure. To investigate the impact of Dpr on aggregation of PrP, we used side chain acetylated FL-PrP 178β-mercapto-Asp-197Dpr(NHAc) under similar conditions as described above. This PrP variant exhibits a similar aggregation tendency as wt FL-PrP, which was analyzed in parallel (Fig. S21†), clearly indicating that PEGylation and not the Asn to Dpr substitution was responsible for affecting the aggregation behavior of PrP. Similar assays performed with PEGylated PrP peptides (aa 178–231) did not show any fibril formation over 60 h (Fig. 3, red circles).

### Homogeneously PEGylated PrP variants inhibit aggregation of unmodified wt PrP

Glycosylation of aggregation-prone proteins such as tau and PrP has been linked to stabilization of secondary structure and a decreased tendency to aggregate.^48,49^ For example, it has recently been shown that *O*-glycosylated tau-derived peptides can selectively inhibit aggregation and toxicity of wild type tau variants.^50^ Triggered by the results of our ThT fluorescence assay, we tested if homogeneously PEGylated PrPs can inhibit fibril formation of unmodified wt PrP. To this end, we mixed wt PrP with homogeneously PEGylated PrP variants in molar ratios of 1 : 1, 5 : 1 and 10 : 1 (Fig. 2A–C), respectively. Remarkably, no increase in ThT fluorescence was observed under these conditions.

Mono- and di-PEGylated PrP peptides have a similar inhibitory effect on fibril formation of wt PrP at ratios of 1 : 1, 1 : 5 and 1 : 10 (Fig. 3A–C), suggesting that interactions in the C-terminal part drive aggregation and fibril formation.^51–53^

## Conclusions

Homogeneous posttranslationally modified PrP variants are essential tools to investigate the possible impacts of PTMs on the pathogenesis of prion diseases. Such studies on PrP can serve as paradigms for other modified proteins involved in neurodegenerative diseases such as tau, α-synuclein and huntingtin. Here, we described a new semisynthetic route that enables access to PrP variants that can be site-specifically modified between residues 178 and 231, thereby covering the two native *N*-glycosylation sites (Asn 181 & 197) as well as the C-terminal GPI attachment site. This strategy was used to successfully introduce PEG_27_ chains as glycan mimics in position 181 and 197. All semisynthetic PrP variants were obtained in amounts of 1.5–1.8 mg and with purities between 90 and 95%. CD spectroscopy demonstrated that site-specifically PEGylated PrP variants are less α-helical than unmodified semisynthetic PrP or wt PrP. Furthermore, in aggregation assays these PEGylated PrP variants showed no tendency to form fibrils under conditions at which unmodified, wt PrP readily forms fibrils. Thus, we have generated homogeneous and site-specifically PEGylated PrP variants, which indicate that modification of *N*-glycosylation sites causes structural alterations that influence aggregation behavior of PrP. Moreover, PEGylation not only prevents fibril formation but also inhibits *in vitro* aggregation of wild type PrP at sub-stoichiometric levels. The latter point gives rise to the question if PrP variants carrying bulky, hydrophilic modifications in positions 181 and 197 (PEG or N-glycans) can form fibrils at all. A similar inhibitory effect is observed for the synthetic PEGylated C-terminal PrP peptides alone, clearly demonstrating their crucial role in PrP aggregation and fibril formation and providing potentially useful inhibitors of these processes.
