# Supplementary material for: Semisynthetic prion protein (PrP) variants carrying glycan mimics at position 181 and 197 do not form fibrils
Source: Chem Sci. 2017 Jul 24;8(9):6626–32. doi: 10.1039/c7sc02719b (PMC5625290; doi:10.1039/c7sc02719b)
Supplement: Supplementary file 1 [file SC-008-C7SC02719B-s001.pdf]

Electronic Supplementary Material (ESI) for Chemical Science.
This journal is © The Royal Society of Chemistry 2017

SUPPLEMENTARY INFORMATION

Semisynthetic Prion Protein (PrP) variants carrying glycan mimics at position 181 and 197 do not form fibrils

Can Araman,[a, b] Robert E. Thompson,[c] Siyao Wang,[c] Stefanie Hackl,[a] Richard J. Payne[c] and Christian F. W. Becker[a,*]

[a] Institute of Biological Chemistry, Department of Chemistry, University of Vienna, Waehringer Strasse 38, 1090

Vienna-AT

[b]Current address: Leiden Institute of Chemistry, Bio-organic Synthesis, Gorlaeus Laboratories, Einsteinweg 55, 2333 CC Leiden-NL

[c] School of Chemistry, The University of Sydney, Sydney, NSW 2006, Australia

* corresponding author, email: christian.becker@univie.ac.at

Table of contents

1. CHARACTERIZATION OF PEGYLATED C-TERMINAL PrP PEPTIDES 4

1.1. PrP 178(β-MERCAPTO-ASP)-181(PEG$_{27}$)-231 4
1.2. PrP 178(β-MERCAPTO-ASP)-197(PEG$_{27}$)-231 4
1.3. PrP 178(β-MERCAPTO-ASP)-181&197(PEG$_{27}$)-231 5

2. CHARACTERIZATION OF ACETYLATED C-TERMINAL PrP PEPTIDES 6

3. GENERAL PROCEDURES FOR INTRODUCTION OF MODIFICATIONS INTO PrP PEPTIDES 8

3.1. *Removal of Mtt(4-methyltrityl) from amino acid side chain.* [1] 8
3.2. *Coupling of Fmoc-NH-(PEG)$_{27}$-OH.* [2] 8
3.3. *Coupling of Boc-Asp(tBu, STmob)-OH to peptidyl-resin.* [3] 8
3.4. *Covalent attachment of acetyl groups into Dpr residues of PrP peptides.* [4] 8

4. GENERAL PROCEDURES FOR RECOMBINANT PROTEIN EXPRESSION 9

4.1. *Recombinant Expression of wild type, full length PrP.* 9

5. EXPRESSED PROTEIN LIGATION (EPL) 9

6. CHARACTERIZATION OF DESULFURIZED, MONO- AND/OR DI-PEGYLATED PrP DERIVATIVES 11

7. CIRCULAR DICHROISM (CD) 11

8. CHARACTERIZATION OF FL-PrP 178(β-MERCAPTO-ASP)-197DPR(NHAC) 17

9. REFERENCES 18

MATERIALS AND METHODS

Chemicals

The following chemicals and solvents were purchased from commercial sources and used without further purification: Fmoc (Fluorenylmethoxycarbonyl)-protected amino acids, 2-(1H-benzotriazol-1-yl)-1,1,3,3-tetramethyl-uronium hexafluorophosphate (HBTU) and 1-[*bis*(dimethylamino)methylene]-1H-1,2,3-triazolo[4,5- b]pyridinium 3-oxid hexafluorophosphate (HATU) (Merck, Darmstadt); succinic anhydride (VWR, Vienna); TentaGel R PHB-Ser(*t*Bu)-Fmoc, TentaGel R PHB-Met-Fmoc (Rapp polymers, Tuebingen); Fmoc-NH-PEG_{27}-COOH (Polypure, Oslo); acetonitrile (ACN), N,N-dimethylformamide (DMF), dichloromethane (DCM), 1,2-dithiothreitol (DTT) (Sigma Aldrich, Vienna) trifluoroaceticacid (TFA) (Biosolve, Netherlands); 4-mercaptophenylaceticacid (MPAA), *tris*(2-carboxyethyl)phosphine (TCEP) (Sigma Aldrich, Vienna). 4-(*tert*-butoxy)-2-((*tert*-butoxycarbonyl)amino)-4-oxo-3-((2,4,5-trimethoxybenzyl)thio)butanoic acid, Boc-Asp(*t*Bu, STmob)-OH, was synthesized as described previously (Thompson, R. E.; Chan, B.; Radom, L.; Jolliffe, K. A.; Payne, R. J. *Angew Chem Int Ed Engl* **2013**, *52*, 9723.). All chemicals and reagents used for buffer preparation were purchased either from Sigma Aldrich (Vienna, Austria) or from VWR (Vienna, Austria). All buffers were prepared with Milli-Q water (ddH_2O).

General methods for Fmoc SPPS

SPPS was performed with Fmoc-protected amino acids (Fmoc-Aa-OH) on Tentagel-MBHA resin. The following side chain protecting groups were used: Arg(Pbf), Asn(Trt), Asp(O*t*Bu), Cys(S*t*Bu), Cys(Trt), Gln(Trt), Glu(O*t*Bu), His(Trt), Lys(Boc), Ser(*t*Bu), Thr(*t*Bu), Trp(Boc) and Tyr(*t*Bu). The non-canonical amino acids Boc-Asp(*t*Bu, STmob)-OH, Fmoc-L-Dpr(Mtt)-OH were used for chemoselective ligation or site selective modification of peptides. Pseudoproline dipeptides Fmoc-Glu-Ser(ψMe,Mepro)-OH, Fmoc-Glu-Thr(ψMe,Mepro)-OH, Fmoc-Ile-Thr(ψMe,Mepro)-OH and Fmoc-Val-Thr(ψMe,Mepro)-OH (Merck, Darmstadt) were used to improve synthesis yields. Peptides were prepared either manually or on automated synthesizers (PTI Tribute synthesizer, Gyros Protein Technologies and Liberty Blue Automated Microwave Synthesizer, CEM, USA). Fmoc deprotection was achieved with 20% piperidine in DMF using two cycles of three and seven minutes. All amino acids (2.5 eq.) were coupled using HBTU or HATU (2.38 eq.) and DIEA (5 eq.) for 30 min, unless stated otherwise. Peptides were globally deprotected and cleaved from dried

resin with a mixture of TFA, triisopropylsilane and water (92.5: 5: 2.5) for 3 h at rt. Precipitation of crude peptides was achieved by addition of three volumes of cold diethyl ether with subsequent centrifugation. After washing twice with ether, precipitated peptides were dissolved in either 5% or 50% ACN in water with 0.1% TFA and lyophilized. All peptides were purified by RP-HPLC using either a Waters Auto Purification HPLC/MS system (3100 Mass Detector, 2545 Binary Gradient Module, 2767 Sample Manager and 2489 UV/Visible Detector) or a Varian ProStar RP-HPLC system. For purification the following reversed phase columns were used: Kromasil 300-10-C4 column (250 × 21.2 mm, 10 µm particle size), Kromasil 300-10-C4 column (250 × 10 mm, 10 µm particle size), Kromasil 300-5-C4 column (250 × 10 mm, 5 µm particle size), Grace Vydac C4 column (250 x 22 mm, 5 µm particle size and a Grace Vydac C4 column (250 x 10 mm, 5 µm particle size). If not indicated otherwise, peptides were dissolved in 6 M Gdn-HCl buffer (pH 4.7) and injected on the reversed phase columns. Elution was achieved by running linear gradients of buffer B (ACN + 0.05 % TFA) in buffer A (ddH2O + 0.05 % TFA). Purified peptides were analyzed by electrospray ionization MS (ESI-MS) operating in positive ion mode. Analytical HPLC analysis of peptides was achieved using a Dionex Ultimate 3000 instrument on a RP-Kromasil-C4-column (300-5-C4, 150 x 4.6 mm, 5 µm particle size) or Thermo Fisher-C4-column (BioBasic-4, 150 x 4.6, 5 µm particle size) at a flow rate of 1 mL/min with a linear gradient from 5 to 65% buffer B (ACN + 0.08% TFA) in buffer A (ddH2O + 0.1% TFA) over 30 min if not stated otherwise. Detection of peptides was at wavelengths of 214 and 280 nm.

Cloning of recombinant PrP 23-177-MxeIntein-H$_6$-CBD.

The plasmid containing this construct was generated from plasmid pTXB3-PrP-23-231-*Mxe*Intein-His$_6$-CBD available in our group. [15]It was modified by an inverse PCR approach with the following primers: forward primer 5´-TGCATCACGGGAGATGCAC-3´, reverse primer 5´-GTGCACGAAGTTGTTCTGG-3´. In doing so, 54 triplets coding for amino acids 178-231 were deleted. The resulting PCR product was purified and digested with DpnI (NEB) overnight (o.n.). After purification, the linearized plasmid DNA was phosphorylated at the 5´ end with T4-Polynucleotide-Kinase PNK (NEB) and ligated with T4-Ligase (NEB) at 22°C for 18 h. The ligation mixture was transformed into *E. coli* XL-1 (Amp^R), grown on an LB-agar-plate (100 µg/mL Ampicillin), colonies were selected and analyzed via colony PCR. Selected clones were sequenced with T7-promotor and T7-terminator primers at Eurofins Genomics AT (Vienna, Austria).

The results were aligned with the theoretical sequence to confirm in-frame fusion of the inserted PrP genes and *Mxe*Intein.

Expression and purification of recombinant PrP 23-177 MESNa α-thioester

All proteins were expressed in 2YT media (16 g/L trypton, 10 g/L yeast extract, 5 g/L NaCl) unless noted otherwise. The plasmid pTXB3-PrP 23-177-*Mxe*Intein-His_6-CBD was transformed into chemically competent Rosetta 2 (DE3) cells (100 µg/mL ampicillin and 30 µg/mL chloramphenicol). Cells were grown until OD_{600nm} 0.6-1.0 and induced with 1 mM isopropyl β-D-1-thiogalactopyranoside (IPTG) for 6 h. Subsequently, cells were harvested by centrifugation (8,900 g, 15 min, and 4°C), resuspended in Tris-buffered saline (TBS; 50 mM Tris-HCl, 150 mM NaCl, pH 8.0) and lysed in a microfluidizer (Constant Systems TS). Lysed cells were centrifuged (50,000 g, 4°C, 30 min) to separate soluble and insoluble components. All analyses performed in this work showed that the PrP-*Mxe*Intein construct was deposited in inclusion bodies (IB). Thus, the pellet was washed with TBS-T buffer (TBS with 0.1% Triton-X, VWR, Vienna) and centrifuged (50,000g, 4°C, 30 min). After three washing and centrifugation cycles with TBS, the pellet was solubilized o.n. in 15 mL of resuspension buffer (8 M Gdn-HCl in TBS, pH 8.0) under continuous stirring. Resuspended fusion protein was centrifuged (50,000g, 4°C, 30 min), the supernatant was collected and loaded on Ni-NTA beads (5 mL, Qiagen) preequilibrated with wash buffer (6 M Gdn-HCl, 50 mM Tris-HCl, pH 8.0) at r.t. After incubation for 30 min, the flow through was collected and Ni-NTA beads were washed with 3 column volumes (CV) of washing buffer with 20 mM imidazole to prevent non-specific binding. Subsequently, the beads were incubated with elution buffer (6 M Gdn-HCl, 50 mM Tris-HCl, 300 mM imidazole, pH 8.0) for 15 min. His-tagged protein was eluted with elution buffer (2 CV) and the column was washed with wash buffer. All elution fractions were combined and concentrated via Amicon Ultra-15 Centrifugal Filter Units (MWCO 30 kDa, VWR, Vienna), the buffer was exchanged to urea buffer (8 M urea, 50 mM TrisHCl pH 8.0) over PD-10 columns (GE Healthcare). The concentration of the resulting protein solution was determined using Nanodrop 2000 (VWR, Vienna) at 280 nm. A solution of 2-mercaptoethanesulfonate (MESNa) in 50 mM Tris-HCl, pH 8.0 was added to the protein solution to reach a final buffer concentration of 4 M urea and protein concentration of ≤ 5 mg/mL. The mixture was gently stirred for 18 h at r.t. to initiate the cleavage of intein. The reaction was analyzed using SDS-PAGE. Upon completion, the reaction was quenched with 6M Gdn-HCl (pH 4.7), concentrated and protein α-thioester was purified over RP-HPLC.

The reaction mixture was loaded on a preparative C4 column (250 x 22 mm, 5 µm particle size, Protein C4, Grace Vydac) that was equilibrated with 5% buffer B (ACN + 0.08% TFA) and 95% buffer A (ddH_2O + 0.1% TFA). A linear gradient of buffer B from 30-90% (v/v) in 60 min was used to elute the protein α-thioesters from the column. Elution fractions containing the protein-thioester were combined; pooled and lyophilized. Subsequent identification was done via analytical RP-HPLC, ESI-MS and SDS-PAGE.

Expressed Protein Ligation (EPL) of recombinant PrP 23-177-MESNa thioester with mono- & di-PEGylated or N-acetylated peptides containing N-terminal β-mercapto-aspartate

All EPL reactions were performed in ligation buffer (6 M Gdn-HCl, 100 mM NaPi buffer and 100 mM MPAA). 50 mM TCEP was used to prevent disulfide formation during EPL. Prior to the ligation, the buffer was degassed with argon for 15 min. Reaction progress was monitored by analytical RP-HPLC and LC-MS. The ligation product was purified by RP-HPLC on a Varian ProStar RP-HPLC system using buffer A and buffer B with a linear gradient of 30-90% B over 60 min. Pure fractions were identified by ESI-MS, analytical RP-HPLC and SDS-PAGE.

Selective desulfurization of β-mercapto-aspartate containing mono- or di-PEGylated PrP

The peptide (2.5 mM) was added to degassed desulfurization buffer (6 M Gdn-HCl, 200 mM NaPi, 250 mM TCEP, 50 mM DTT, adjusted to pH 2.8-3.0) as a solid. The solution was flushed with argon for 5 min and the reaction vessel was incubated at 65°C for at least 7 h. The reaction was monitored by analytical RP-HPLC and LC-MS. Upon completion, the reaction mixture was quenched with a four-fold excess of 6 M Gdn-HCl (pH 4.7) and immediately purified by RP-HPLC on a Varian ProStar RP-HPLC System using buffer A and buffer B with a linear gradient of 30-90% B over 60 min. Product containing collected fractions were pooled, lyophilized and stored at -80°C for further use.

Folding of non-, mono-, di-PEGylated and acetylated PrP variants

Based on the procedure by Chu *et al.* all acetylated, PEGylated and non-PEGylated PrP variants were folded: lyophilized PrP variants were dissolved in denaturing buffer (6 M Gdn-HCl, 50 mM Tris-HCl, pH 8.0) and diluted stepwise to 2 M Gdn-HCl with folding buffer (20 mM Na-acetate, 0.3/3 mM GSSG/GSH, pH 5.0) at 4°C. The reaction mixture was incubated at 4°C for 2 d while being dialyzed against refolding buffer (500-

fold sample volume), first for 3 h then overnight. The resulting protein solution was centrifuged at 14,000 g at 4°C for 15 min to remove insoluble PrP. Folded protein was analyzed using a NanoDrop 2000 (VWR, Vienna, Austria) to determine concentrations (typically between 0.05-0.2 mg/mL) and CD spectra were collected.

Circular Dichroism (CD)

Far UV-CD spectra were measured using a Chirascan Plus spectrometer (Applied Photophysics, UK) between 190-260 nm. Unless stated otherwise, ten spectra with an acquisition time of 10 s for each scan in a 1 mm quartz cell at 1 nm resolution were acquired at r.t. and averaged. Typical protein concentrations were 0.1 mg/mL in folding buffer. To determine the secondary structure, minima at 208 and 222 nm as well as maxima at 193 nm for α-helices were considered, whereas a negative peak at 218 nm and a positive peak at 195 nm were used for the calculation of the percentage of anti-parallel β-sheets. The amount of random coil structure was calculated using the maximum peak at 210 nm and minimum peak at 195 nm. All calculations were carried out using CDNN software comparing at least 13 spectra from the database.

Aggregation assays with Thioflavin-T (ThT)

For aggregation assays a procedure modified from Baskakov *et al.* was used, 1 mM ThT in ddH_2O and 2 M Gdn-HCl were added to a solution of folded, PEGylated and non-PEGylated PrP variants at a concentration of 0.1 mg/mL in folding buffer. The pH was adjusted to 6.0 and three 160 µl samples were pipetted into three separate wells of a 96 well plate. To allow constant mixing and to prevent evaporation, a single, sterile glass bead (Ø= 2 mm, Merck) was placed into the well and the plate was covered with a sealer. The plate was incubated at 37°C under continuous agitation using a microplate reader (Biotek Synergy Mx or Tecan Infinite M1000 Pro for data shown in Fig. S21). ThT fluorescence was recorded every 10 min with an excitation wavelength of 444±9 nm and an emission wavelength of 485±9 nm for 60 hours.

1. Characterization of PEGylated C-terminal PrP peptides

1.1. *PrP 178(β-mercapto-Asp)-181(PEG_{27})-231*

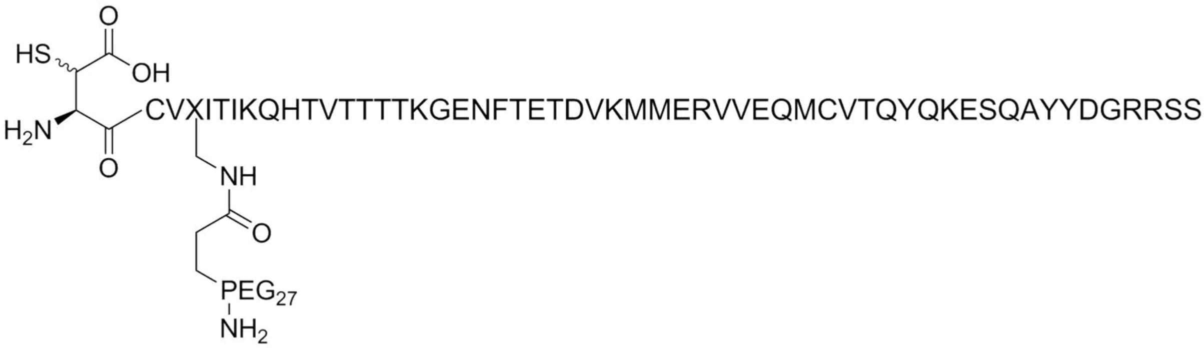

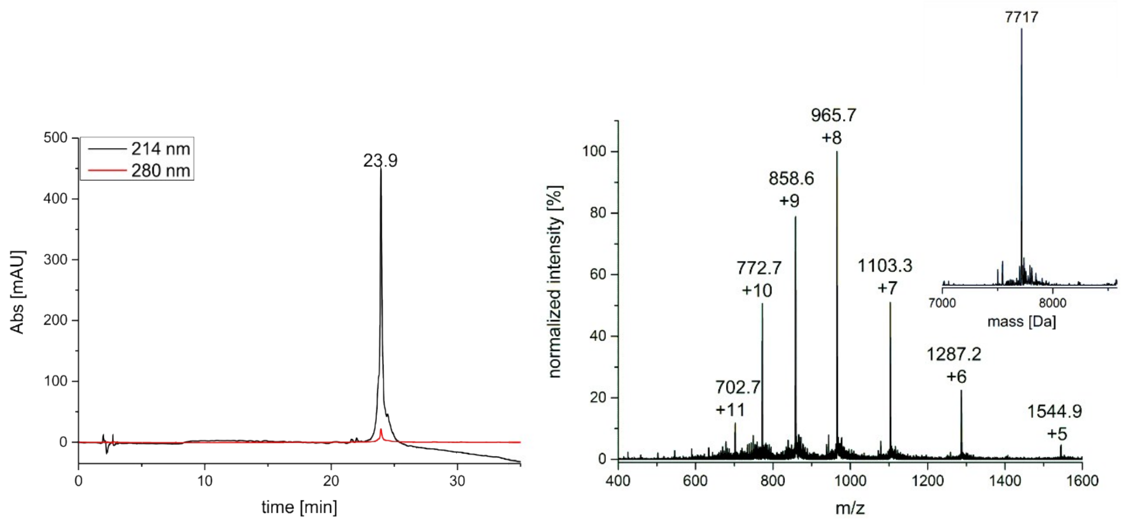

Figure S1: Characterization of purified PrP 178(β-mercapto-Asp)-181(PEG_{27})-231.
Left panel: analytical C4 RP-HPLC chromatogram of purified PrP 178(β-mercapto-Asp)-181(PEG_{27})-231, linear gradient 5-65% ACN in 30 min. Right panel: ESI-MS spectrum of purified PrP 178(β-mercapto-Asp)-181(PEG_{27})-231, expected mass: 7718.8 Da, observed mass: 702.7 $[M+11H]^{11+}$, 772.7 $[M+10H]^{10+}$, 858.6 $[M+9H]^{9+}$, 965.7 $[M+8H]^{8+}$, 1103.3 $[M+7H]^{7+}$, 1287.2 $[M+6H]^{6+}$, 1544.9 $[M+5H]^{5+}$. The deconvoluted spectrum showed a mass of 7717 Da

1.2. *PrP 178(β-mercapto-Asp)-197(PEG_{27})-231*

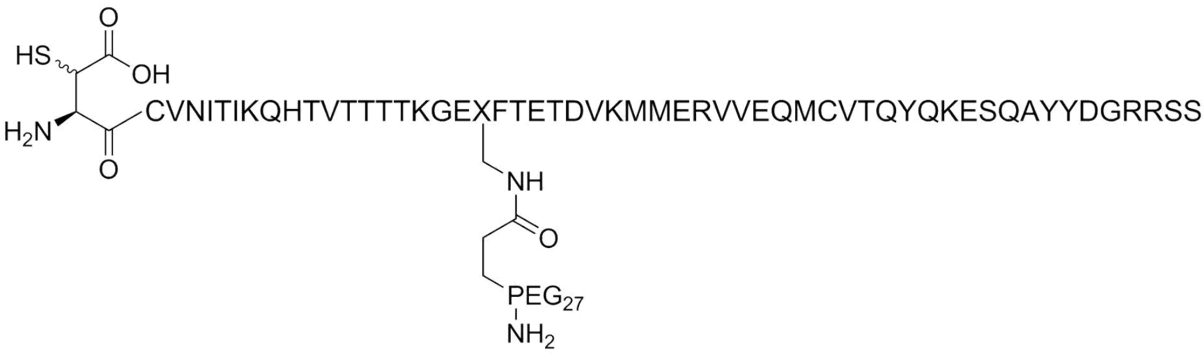

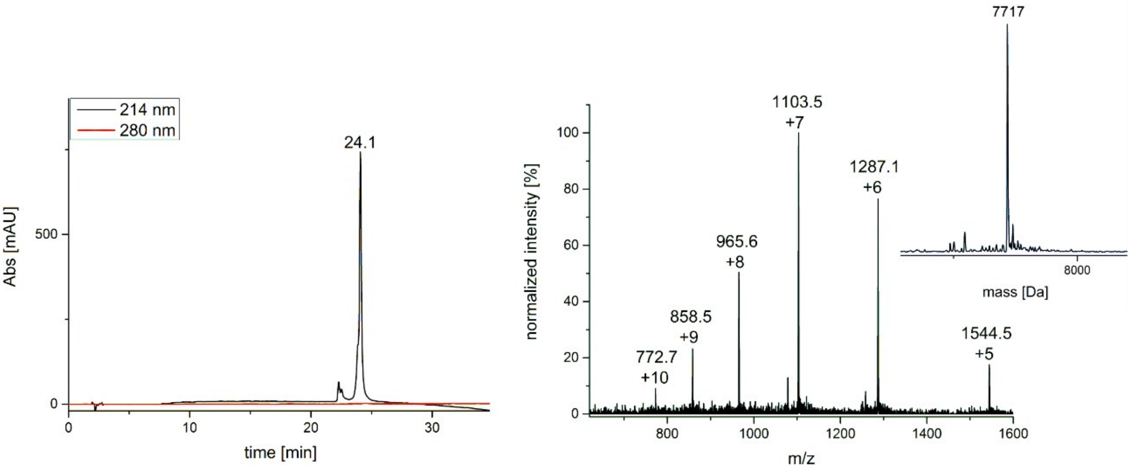

Figure S1. Characterization of purified PrP 178(β-mercapto-Asp)-197(PEG_{27})-231.
Left panel: analytical C4 RP-HPLC chromatogram of purified PrP 178(β-mercapto-Asp)-197(PEG_{27})-231, linear gradient 5-65% ACN in 30 min. Right panel: ESI-MS spectrum of purified PrP 178(β-mercapto-Asp)-181(PEG_{27})-231, expected mass: 7718.8 Da, observed mass: 772.7 [M+10H]10+, 858.5 [M+9H]9+, 965.6 [M+8H]8+, 1103.5 [M+7H]7+, 1287.1 [M+6H]6+, 1544.5 [M+5H]5+. The deconvoluted spectrum showed a mass of 7717 Da.

1.3. *PrP 178(β-mercapto-Asp)-181&197(PEG_{27})-231*

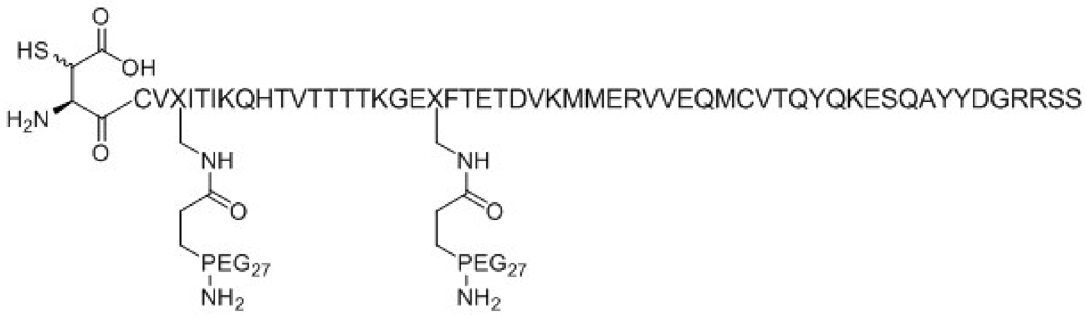

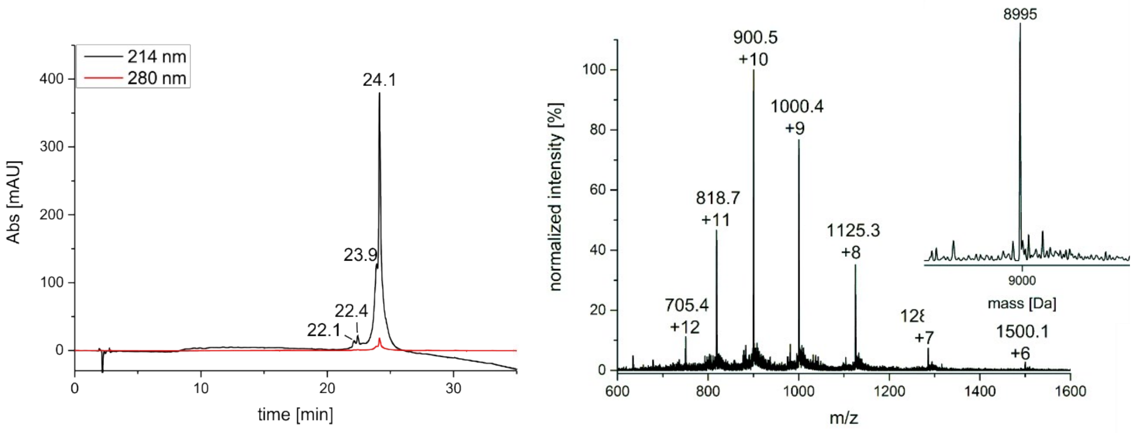

Figure S3. Characterization of purified PrP 178(β-mercapto-Asp)-181&197(PEG_{27})-231.
Left panel: analytical C4 RP-HPLC chromatogram of purified PrP 178(β-mercapto-Asp) 181&197(PEG27)-231, linear gradient 5-65% ACN in 30 min. Right panel: ESI-MS spectrum of purified PrP 178(β-mercapto-Asp)-181(PEG_{27})-231, expected mass: 8995.3 Da, observed mass: 705.4 [M+12H]12+, 818.7 [M+11H]11+, 900.5 [M+10H]10+, 1000.4 [M+9H]9+, 1125.3 [M+8H]8+, 1285.9 [M+7H]7+, 1500.1 [M+6H]6+. The deconvoluted spectrum showed a mass of 8995 Da.

Table 1: Overall yields of side chain PEGylated PrP peptides.

variant	***amount***	***overall yield***
PrP 178-(β-mercapto-Asp)-181PEG$_{27}$-231	43.0 mg	22%
PrP 178-(β-mercapto-Asp)-197PEG$_{27}$-231	42.3 mg	22%
PrP 178-(β-mercapto-Asp)-181&197PEG$_{27}$-231	33.7 mg	15%

2. Characterization of acetylated C-terminal PrP peptides

2.1. *PrP 178(β-mercapto-Asp)-181Dpr(NHAc)-231*

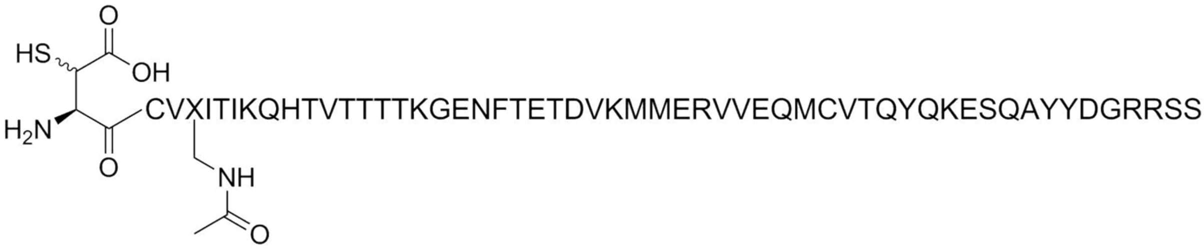

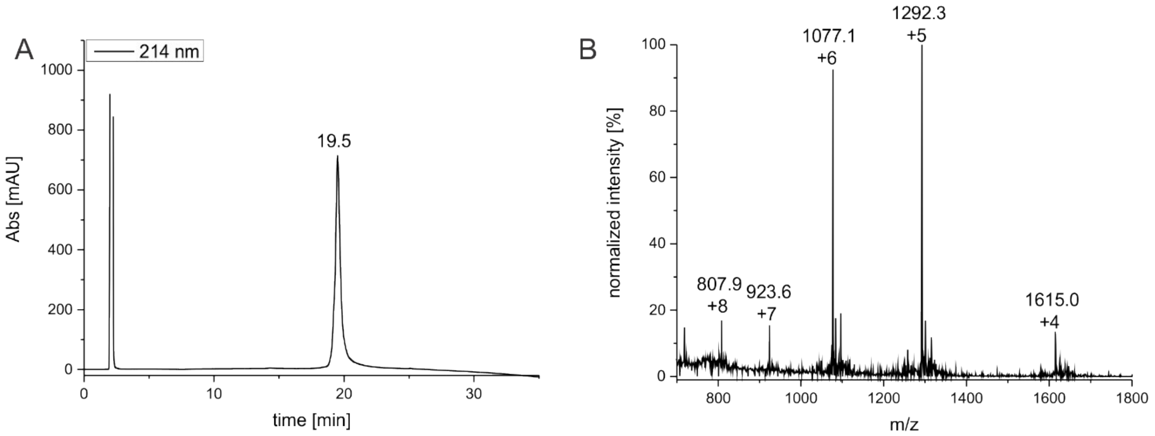

Figure S4: Characterization of purified PrP 178(β-mercapto-Asp)-181Dpr(NHAc)-231.
A: analytical C4 RP-HPLC chromatogram of purified PrP 178(β-mercapto-Asp)-181Dpr(NHAc)-231, linear gradient 5-65% ACN in 30 min. Right panel: ESI-MS spectrum of purified PrP 178(β-mercapto-Asp)-181Dpr(NHAc)-231, expected mass: 6456.5 Da, observed mass: 6456.4; 807.9 $[M+8H]^{8+}$, 923.6 $[M+7H]^{7+}$, 1077.1 $[M+6H]^{6+}$, 1292.3 $[M+5H]^{5+}$, 1615.0 $[M+4H]^{4+}$.

2.2. *PrP 178(β-mercapto-Asp)-197(NHAc)-231*

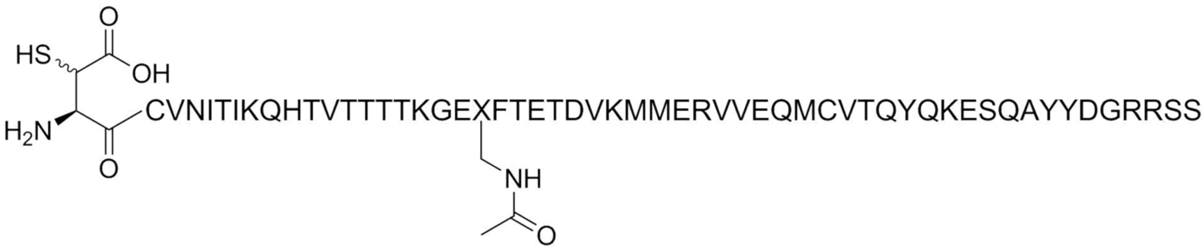

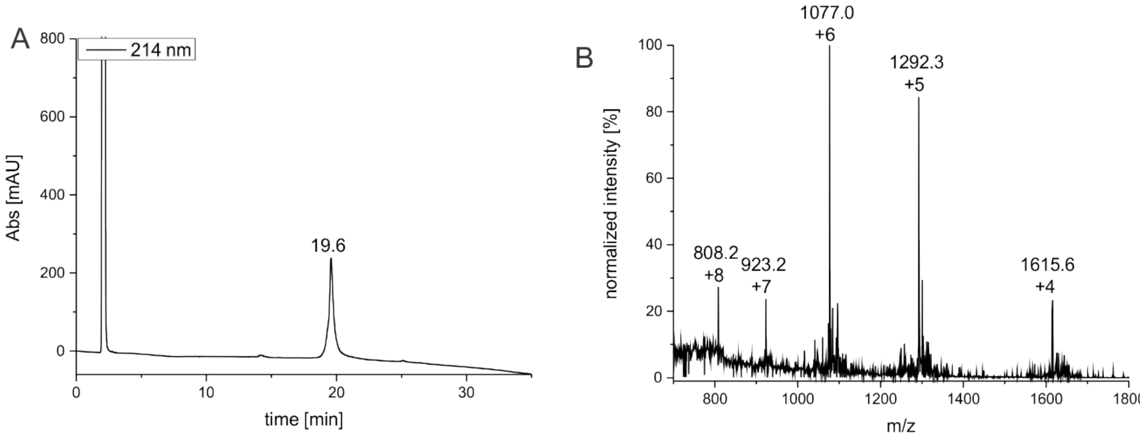

Figure S5: Characterization of purified PrP 178(β-mercapto-Asp)-197Dpr(NHAc)-231.
A: analytical C4 RP-HPLC chromatogram of purified PrP 178(β-mercapto-Asp)-197Dpr(NHAc)-231, linear gradient 5-65% ACN in 30 min. Right panel: ESI-MS spectrum of purified PrP 178(β-mercapto-Asp)-197Dpr(NHAc)-231, expected mass: 6456.5 Da, observed mass: 6456.8; 808.2 $[M+8H]^{8+}$, 923.2 $[M+7H]^{7+}$, 1077.0 $[M+6H]^{6+}$, 1292.3 $[M+5H]^{5+}$, 1615.6 $[M+4H]^{4+}$.

2.3. *PrP 178(β-mercapto-Asp)-181&197(NHAc)-231*

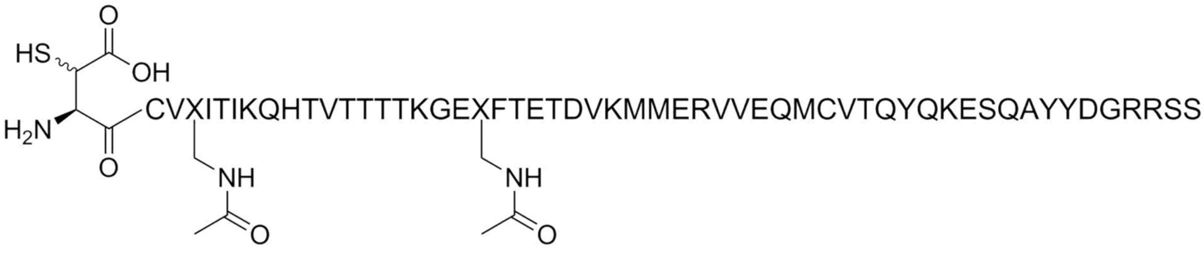

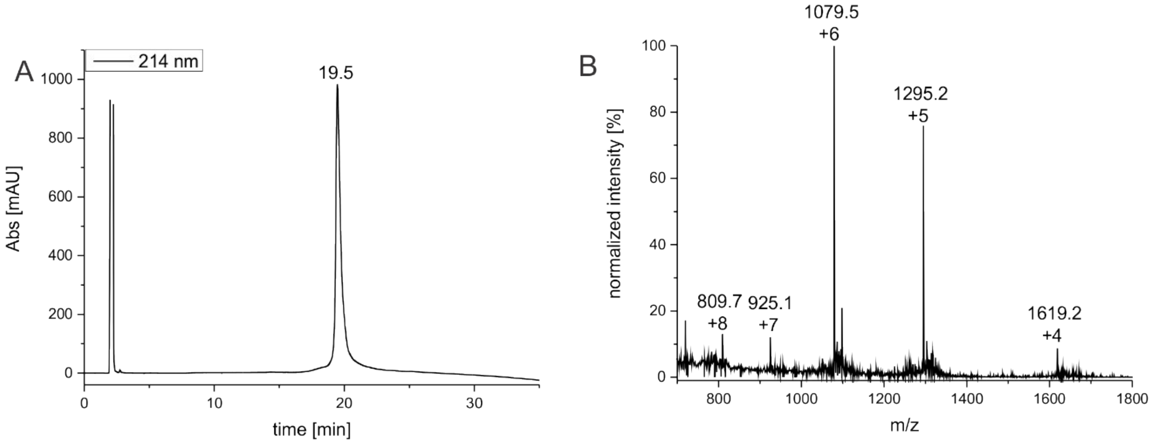

Figure S6: Characterization of purified PrP 178(β-mercapto-Asp)-181&197Dpr(NHAc)-231.
A: analytical C4 RP-HPLC chromatogram of purified PrP 178(β-mercapto-Asp)-181&197Dpr(NHAc)-231, linear gradient 5-65% ACN in 30 min. Right panel: ESI-MS spectrum of purified PrP 178(β-mercapto-Asp)-181&197Dpr(NHAc)-231, expected mass: 6470.2 Da, observed mass: 6470.6; 809.7 $[M+8H]^{8+}$, 925.1 $[M+7H]^{7+}$, 1079.5 $[M+6H]^{6+}$, 1295.2 $[M+5H]^{5+}$, 1619.2 $[M+4H]^{4+}$.

Table 2: Overall yields of side chain acetylated PrP peptides.

variant	*amount*	*overall yield*
PrP 178-(β-mercapto-Asp)-181NHAc-231	20.1 mg	25%
PrP 178-(β-mercapto-Asp)-197NHAc-231	16.2 mg	20%
PrP 178-(β-mercapto-Asp)-181&197NHAc-231	32.0 mg	40%

3. General procedures for introduction of modifications into PrP peptides

3.1. *Removal of Mtt(4-methyltrityl) from amino acid side chain.* [1]

The peptidyl resin was washed with DCM and swollen in DCM for 1-3 h. A solution of 98% DCM, 1% TIS, 1% TFA was added to the peptidyl resin and agitated for 2 min giving rise to a yellow color. After washing of the peptidyl resin with DCM, this procedure was repeated until the yellowish color vanished upon addition of the DCM/TIS/TFA solution. For 0.05 mmol peptidyl resin, roughly 150 mL deprotection solution was applied. The resin was washed with DMF and swollen for 2 h in DMF prior to further synthesis.

3.2. *Coupling of Fmoc-NH-(PEG)$_{27}$-OH.* [2]

Fmoc-NH-(PEG)$_{27}$-OH (2.75 eq.) was dissolved in a solution of 0.5 M HATU in ACN/DMF (40:60 v/v) (2.5 eq. HATU) and 5 eq. DIEA was added. The solution was transferred into the syringe with the peptidyl resin and the syringe was rotated for 20 h. The resin was washed with DMF and subsequently with DCM.

3.3. *Coupling of Boc-Asp(tBu, STmob)-OH to peptidyl-resin.* [3]

Boc-Asp(*t*Bu, STmob)-OH (2.0 eq.) was dissolved in a mixture of PyBOP (2.0 eq.), and NMM (4.0 eq.) in DMF (final concentration 0.1 M). The reaction mixture was then added to the resin (1.0 eq.) and rotated at rt for 20 h. Subsequently, the resin was washed with DMF (5 x 3 mL per g resin), DCM (5 × 3 mL per g resin), DMF (5 × 3 mL per g resin), and DCM (10 × 3 mL per g resin).

3.4. *Covalent attachment of acetyl groups into Dpr residues of PrP peptides.* [4]

Mtt temporary protecting groups on Dpr amine side chains were deprotected as described in 3.1. The N-terminus of the peptidyl resin was Boc protected. Acetic anhydride (20% v/v) and DIEA (20% v/v) was added to a mixture of DMF/DCM (1/1). This mixture (10 mL/g resin) was added to the peptidyl resin and reacted for 1 h. Upon completion, the resin was washed with DMF (3 mL, 1min) and dried *in vacuo.*

4. General procedures for recombinant protein expression

4.1. *Recombinant Expression of wild type, full length PrP*.

Wild type (wt), recombinant PrP was generated via hydrolysis of recombinant, full length PrP α-thioester as described previously by Becker and coworkers. [5]

5. Expressed Protein Ligation (EPL)

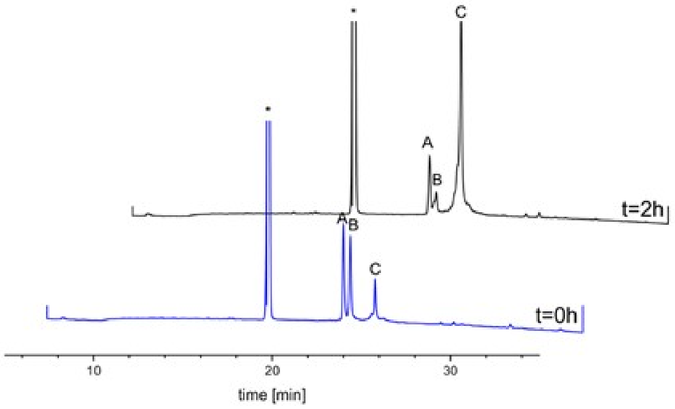

Figure S7. Ligation of PrP 23-177-MESNA thioester with 178(β-mercapto-Asp)-181-PEG$_{27}$-231.
A: PrP 23-177 α-thioester, B: PrP 178(β-mercapto-Asp)-181PEG$_{27}$-231, C: ligation product+MPAA. The reaction was monitored during 2 h of reaction time. *: MPAA. Chromatograms were recorded on a C4 column with a linear gradient 5-65% ACN in 30 min.

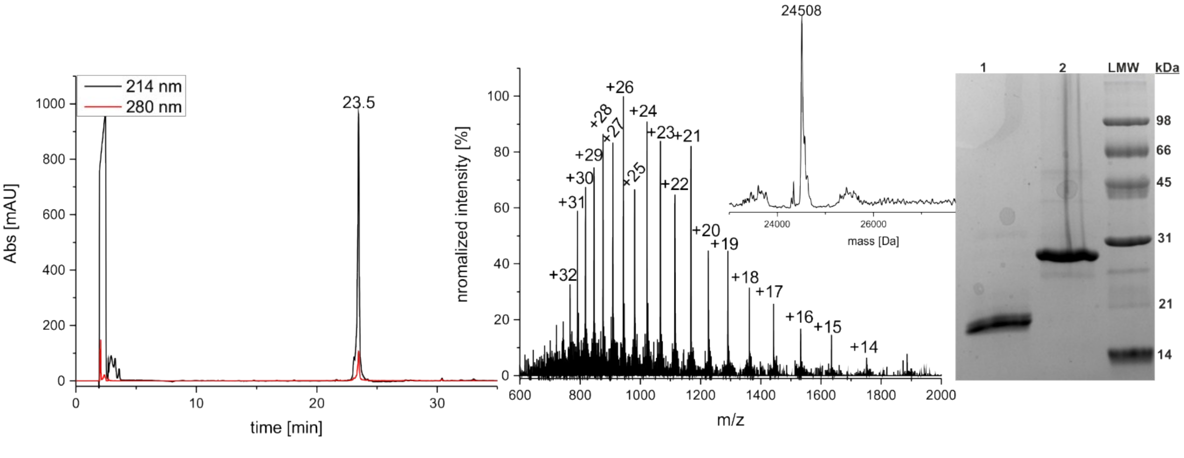

Figure S8. Characterization of PrP 23-178 β-mercapto-Asp-181PEG$_{27}$-231, expected mass: 24509.4 Da, observed mass: 24508 Da. The final product was analyzed via analytical RP-HPLC, ESI-MS and SDS-PAGE.

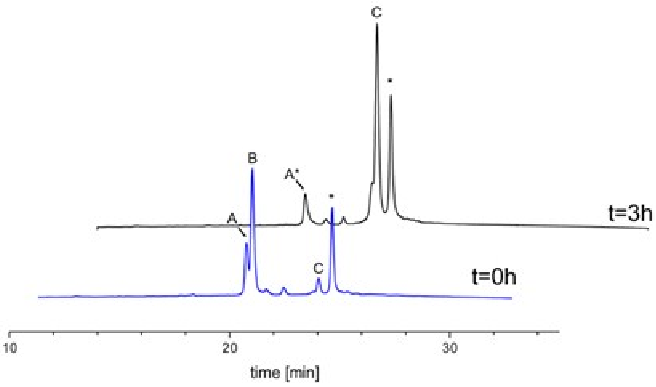

Figure S9. Ligation of PrP 23-177-MESNA thioester with 178(β-mercapto-Asp)-197-PEG$_{27}$-231.
A: PrP 23-177 α-thioester, B: PrP 178(β-mercapto-Asp)-197PEG27-231, C: ligation product. The reaction was monitored during 3 h of reaction time. *: MPAA, A*: hydrolyzed PrP 23-177-OH. Chromatograms were recorded on a C4 column with a linear gradient 5-65% ACN in 30 min.

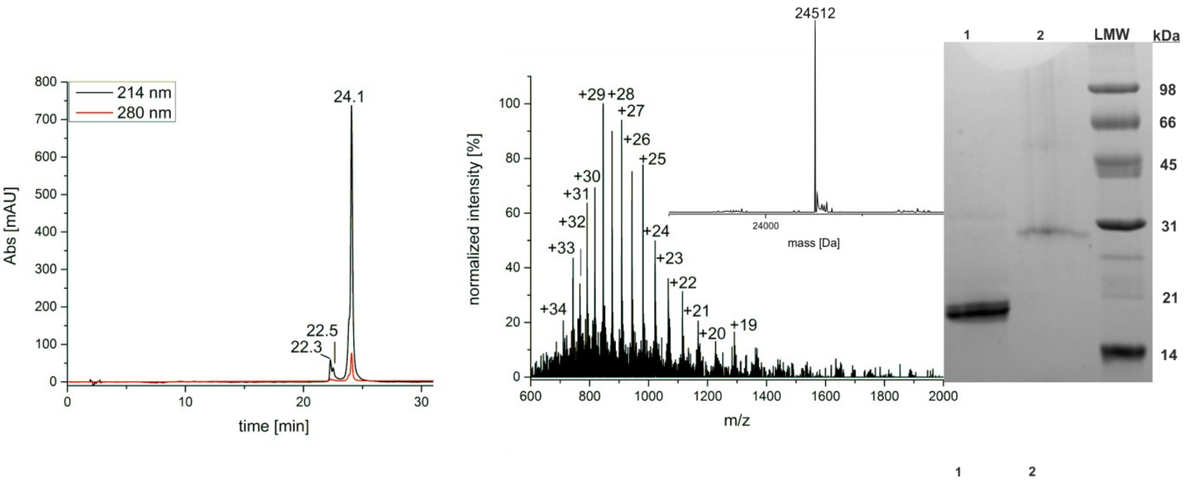

Figure S10. Characterization of PrP 23-178 β-mercapto-Asp-197PEG$_{27}$-231, expected mass: 24509.4 Da, observed mass: 24512 Da. The final product was analyzed via analytical RP-HPLC, ESI-MS and SDS-PAGE.

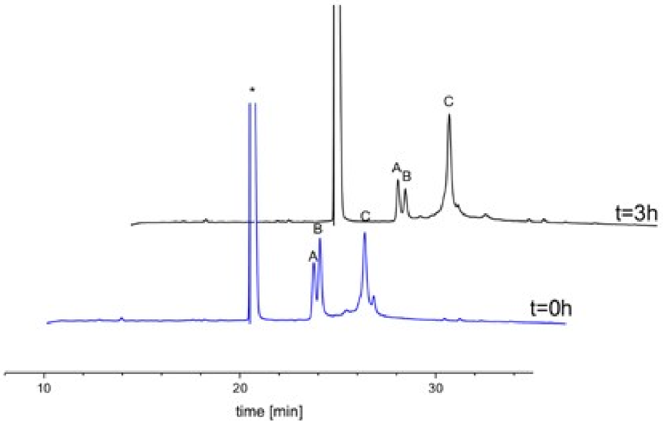

Figure S11. Ligation of PrP 23-177-MESNA thioester with 178(β-mercapto-Asp)- 181&197PEG$_{27}$-231.
A: PrP 23-177 α-thioester and PrP 23-177-OH, B: PrP 178(β-mercapto-Asp)-181&197PEG$_{27}$-231, C: ligation product. The reaction was monitored during 3 h of reaction time. *: MPAA. Chromatograms were recorded on a C4 column with a linear gradient of 5-65% ACN in 30 min.

6. Characterization of desulfurized, mono- and/or di-PEGylated PrP derivatives

6.1. PrP 23-181PEG$_{27}$-231

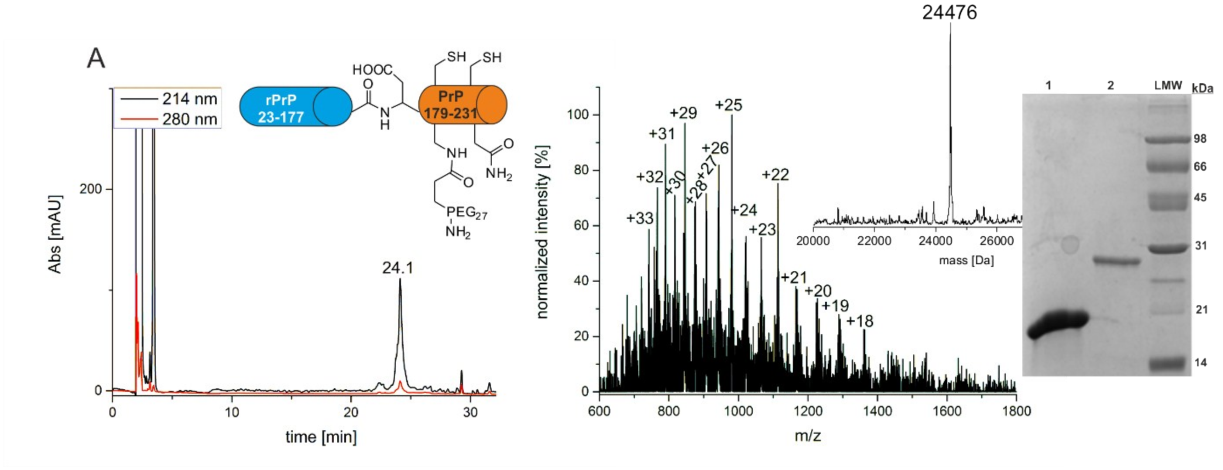

Figure S12. Characterization of PrP 23-181PEG$_{27}$-231. The final product was analyzed via analytical RP-HPLC, ESI-MS (expected mass: 24476.6 Da, observed mass: 24476 Da) and SDS-PAGE (lane 1: PrP 23-177-MESNA thioester, lane 2: PrP 23-181PEG$_{27}$-231).

6.2. PrP 23-197PEG$_{27}$-231

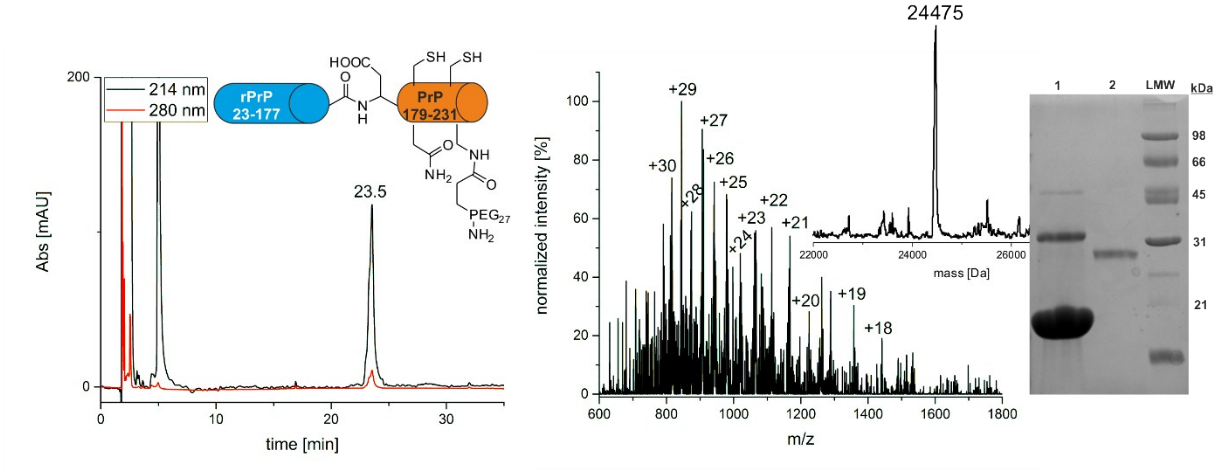

Figure S13. Characterization of PrP 23-197PEG$_{27}$-231. The final product was analyzed via analytical RP-HPLC, ESI-MS (expected mass: 24476.6 Da, observed mass: 24475 Da) and SDS-PAGE (lane 1: PrP 23-177-MESNA thioester, lane 2: PrP 23-197PEG$_{27}$-231).

7. Circular Dichroism (CD)

All PEGylated and N-acetylated peptides and proteins were folded as follows; Lyophilized, N-acetylated peptides were dissolved in 6 M Gdn-HCl in 50 mM Tris (pH 8.0) and diluted to 2 M Gdn-HCl stepwise with folding buffer (20 mM Sodium acetate,

0.3/3 GSSG/GSH, pH 5.0). Subsequently all samples were dialyzed against 20 mM sodium acetate (pH 5.0), first 3 h and then overnight (o.n.). Upon concentration determination, CD data was recorded using a Chirascan Plus spectrometer (Applied Photo-physics, UK) within a wavelength range of 190-260 nm. Typical concentrations were 0.08-0.1 mg/mL. [5]

Lyophilized, PEGylated peptides were dissolved in folding buffer and incubated at 4°C o.n. All samples were dialyzed against 20 mM sodium acetate buffer (pH 5.0) and CD data was recorded. Typical concentrations were 0.1-0.3 mg/mL.

7.1. Determination of secondary structure of homogeneously PEGylated FL-PrP variants

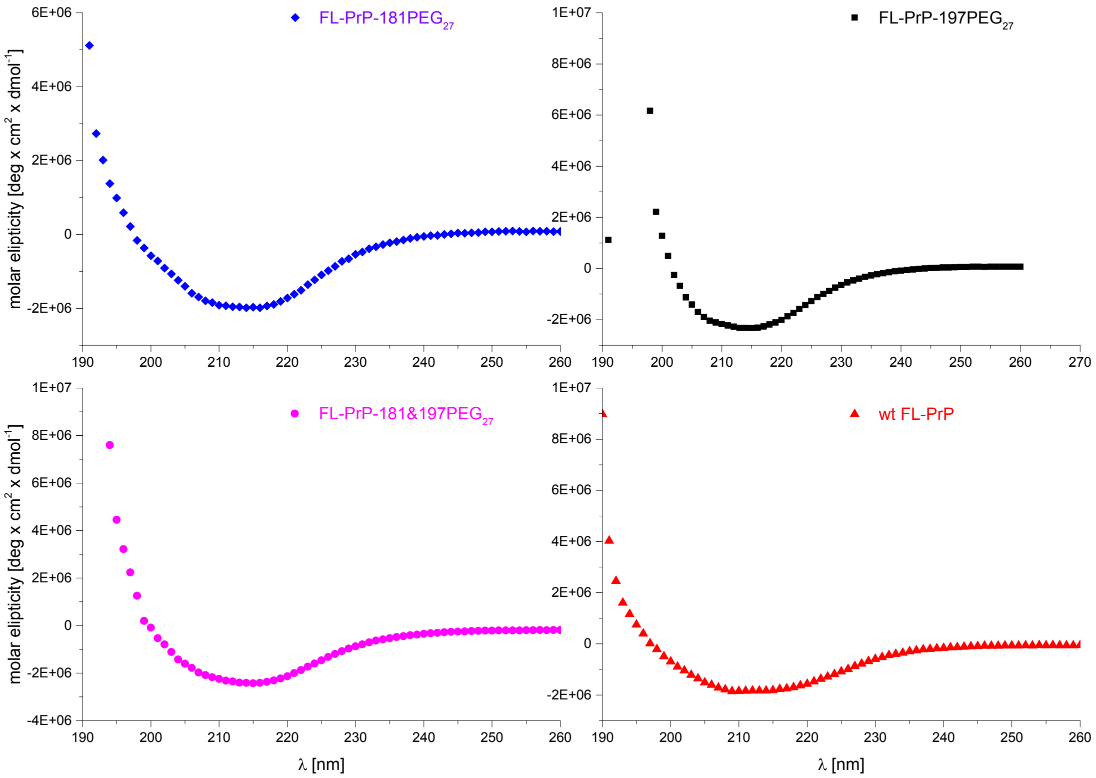

Figure S14. Biophysical characterization of PEGylated PrP variants via circular dichroism (CD). Secondary structure determination via CD spectroscopy. All data was recorded within the range of 190nm-260nm.

7.2. Determination of secondary structure for Dpr side chain N-acetylated PrP peptides

7.2.1. PrP 178(β-mercapto-Asp)-181NHAc-231

The secondary structure was determined using CDNN software via normalization of at least 13 spectra.

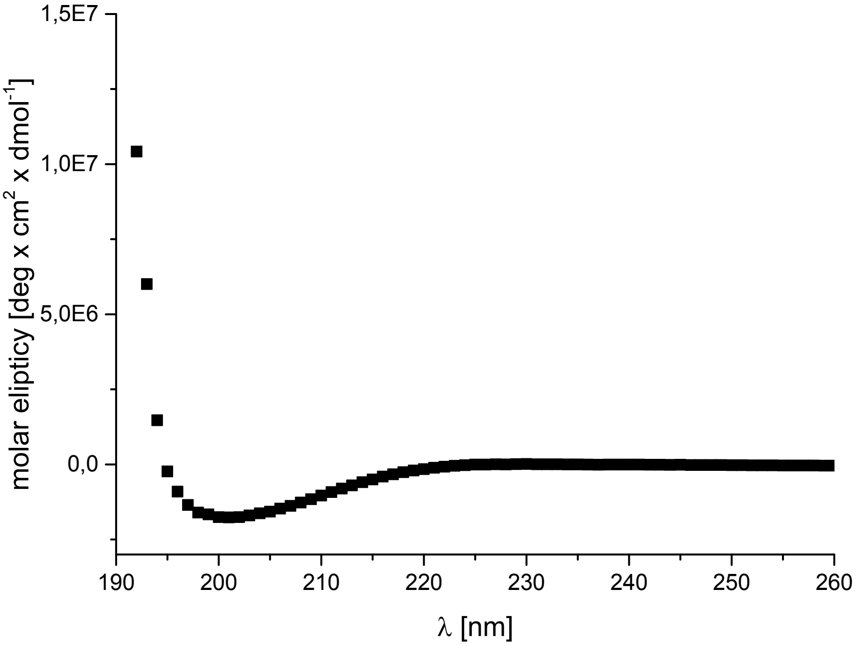

Figure S15. CD spectrum of PrP 178(β-mercapto-Asp)-181NHAc-231.

PrP 178(β-mercapto-Asp)-181NHAc-231 showed a minimum at 200 nm, which corresponds to a high content of random coil. Additionally, positive values below 195 nm highlighted a tendency for β-sheets, whereas the typical minimum at 218 was not observed, suggesting a predominant random coiled structure (Figure S10).

7.2.2. PrP 178(β-mercapto-Asp)-197NHAc-231

Similar results to PrP 178(β-mercapto-Asp)-181NHAc-231 were obtained (Figure S11). These results suggest that regardless of the position of acetylation, Dpr side chain acetylated C-terminal PrP peptides do not fold into a native full length-like structure.

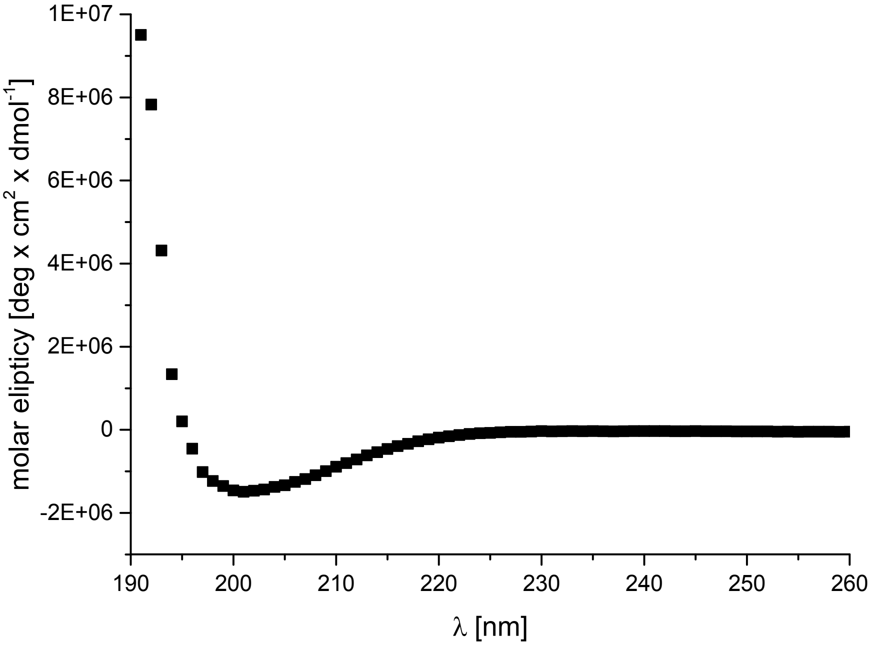

Figure S16. CD spectrum of PrP 178(β-mercapto-Asp)-197NHAc-231.

7.3. Determination of secondary structure of mono- and di-PEGylated PrP peptides

Mono- and di-PEGylated PrP with N-terminal ß-mercapto-Asp was refolded as described in section 6.1. Unlike its N-acetylated counterpart, a defined structure for PrP 178ß-mercapto-Asp-XPEG$_{27}$-231 (X= aa 181 and/or 197) was observed. Moreover, compared to the CD spectrum of PrP 23-XPEG$_{27}$-231 (X= aa 181 and/or 197), these peptides showed a higher α-helical content (Figure S12-14).

7.3.1. PrP 178(β-mercapto-Asp)-181PEG$_{27}$-231

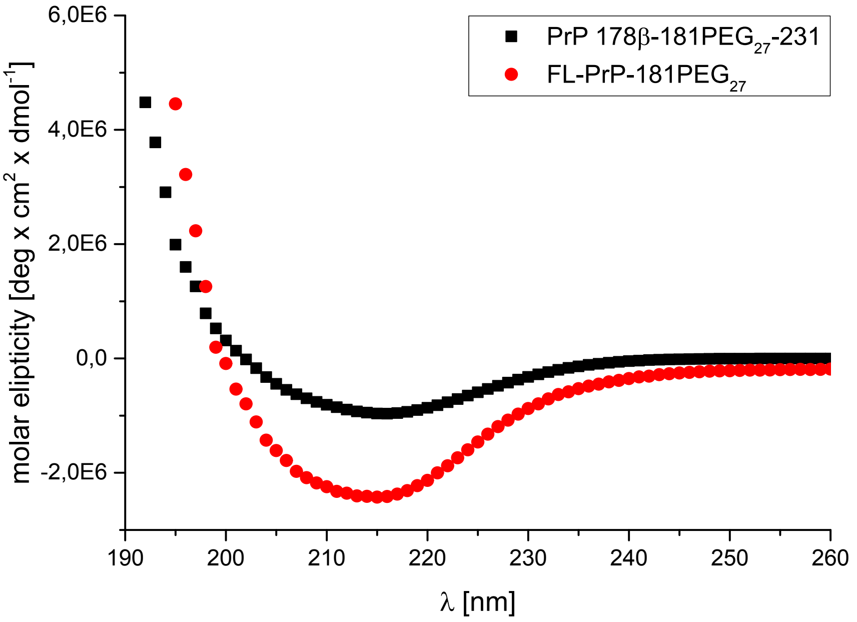

Figure S17. CD spectra of PrP 178(β-mercapto-Asp)-181PEG$_{27}$-231 (black squares) and FL-PrP-181PEG$_{27}$ (red dots).

7.3.2. PrP 178(β-mercapto-Asp)-197PEG$_{27}$-231

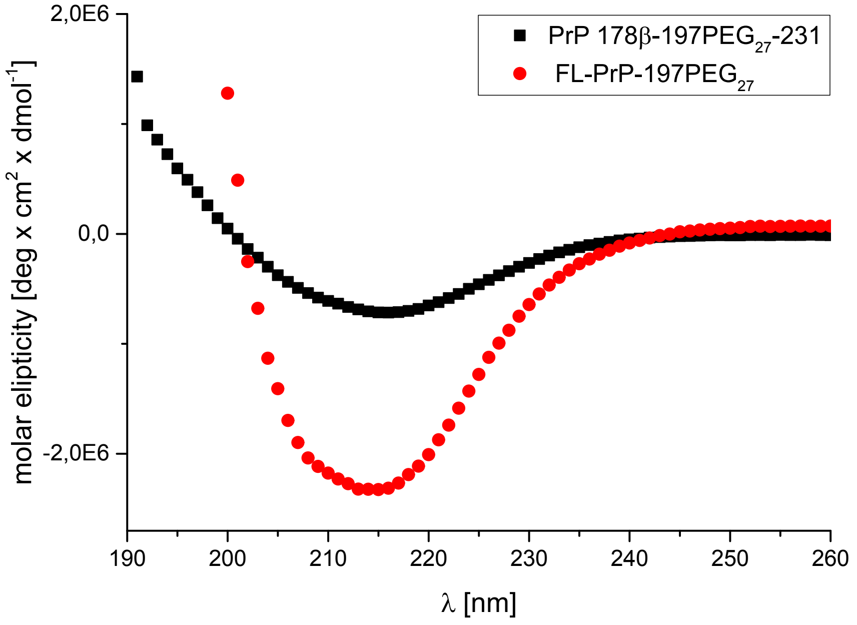

Figure S18. CD spectra of PrP 178(β-mercapto-Asp)-197PEG$_{27}$-231 (black squares) and FL-PrP-197PEG$_{27}$ (red dots).

7.3.3. PrP 178(β-mercapto-Asp)-181&197PEG$_{27}$-231

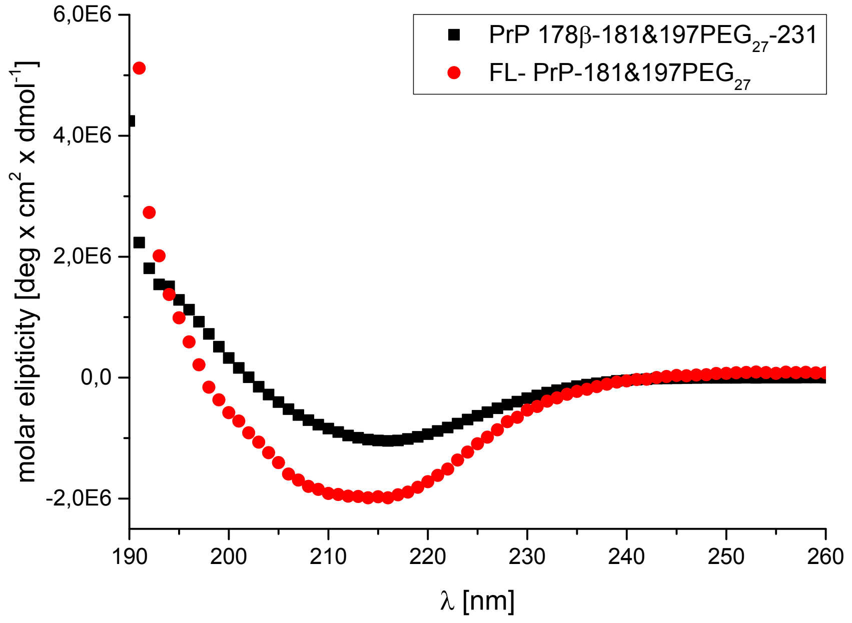

Figure S19. CD spectra of PrP 178(β-mercapto-Asp)-181&197PEG$_{27}$-231 (black squares) and FL-PrP-181&197PEG$_{27}$ (red dots).

7.4. Determination of the secondary structure of FL-PrP 178(β-mercapto-Asp)-197Dpr(NHAc)

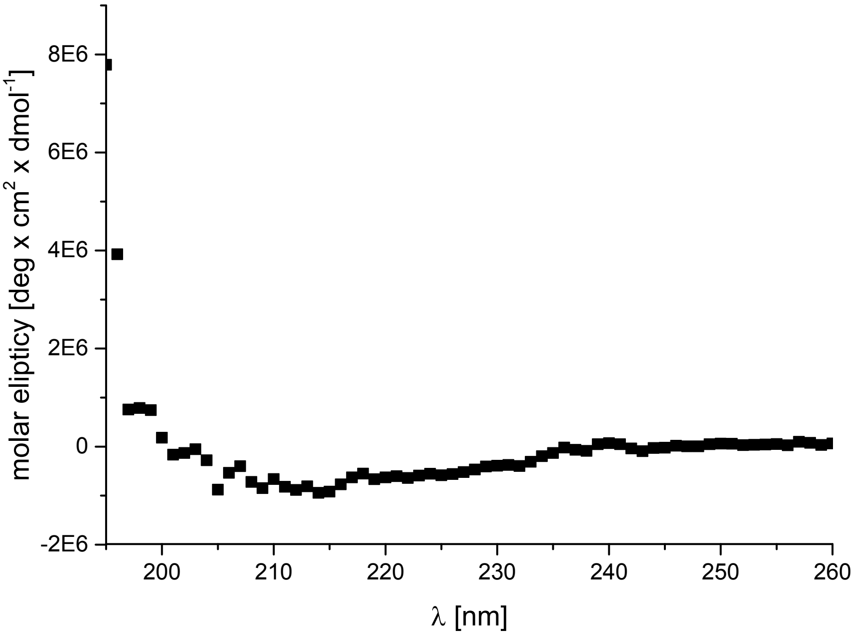

Figure S20. CD spectrum of FL-PrP-178(β-mercapto-Asp)-197Dpr(NHAc)

8. Characterization of FL-PrP 178(β-mercapto-Asp)-197Dpr(NHAc)

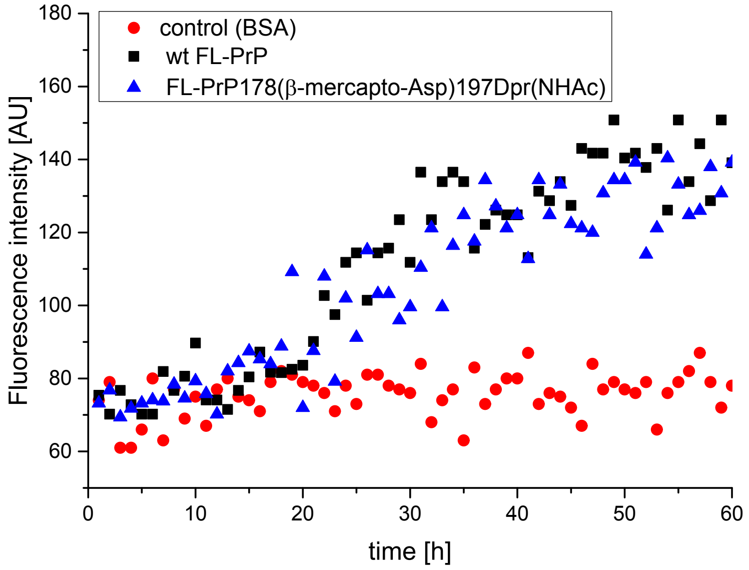

Figure S21. ThT Fluorsecence aggregation assay of wt FL-PrP (black squares) and FL-PrP 178(β-mercapto-Asp)-197Dpr(NHAc) (blue triangles) in comparison to BSA as a negative control (red circles).

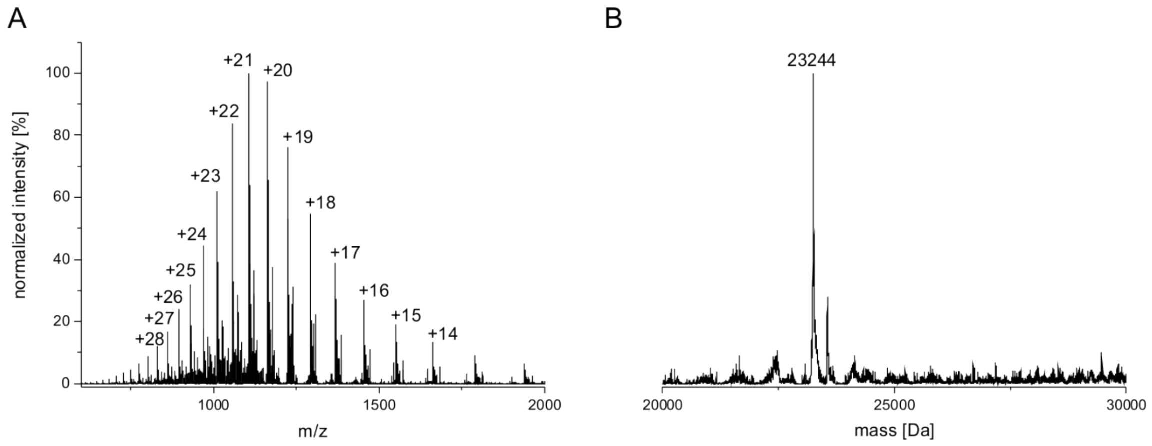

Figure S22. Characterization of FL-PrP 178(β-mercapto-Asp)-197Dpr(NHAc) mass spectrometry. A: ESI-MS of FL-PrP 178(β-mercapto-Asp)-197Dpr(NHAc). B: Deconvoluted mass spectrum of FL-PrP 178(β-mercapto-Asp)-197Dpr(NHAc) (calculated mass: 23244 Da, observed mass: 23244 Da).

.

9. References

(1) Aletras, A.; Barlos, K.; Gatos, D.; Koutsogianni, S.; Mamos, P. *International Journal of Peptide and Protein Research* **1995**, *45*, 488.

(2) Bello, C.; Wang, S.; Meng, L.; Moremen, K. W.; Becker, C. F. *Angew Chem Int Ed Engl* **2015**, *54*, 7711.

(3) Thompson, R. E.; Chan, B.; Radom, L.; Jolliffe, K. A.; Payne, R. J. *Angew Chem Int Ed Engl* **2013**, *52*, 9723.

(4) Pennington, M. W. In *Peptide Synthesis Protocols*; Pennington, M. W., Dunn, B. M., Eds.; Humana Press: Totowa, NJ, 1995, p 171.

(5) Chu, N. K.; Shabbir, W.; Bove-Fenderson, E.; Araman, C.; Lemmens-Gruber, R.; Harris, D. A.; Becker, C. F. *J Biol Chem* **2014**, *289*, 30144.
